# Current landscape of mRNA technologies and delivery systems for new modality therapeutics

**DOI:** 10.1186/s12929-024-01080-z

**Published:** 2024-09-10

**Authors:** Ruei-Min Lu, Hsiang-En Hsu, Ser John Lynon P. Perez, Monika Kumari, Guan-Hong Chen, Ming-Hsiang Hong, Yin-Shiou Lin, Ching-Hang Liu, Shih-Han Ko, Christian Angelo P. Concio, Yi-Jen Su, Yi-Han Chang, Wen-Shan Li, Han-Chung Wu

**Affiliations:** 1 Biomedical Translation Research Center, Academia Sinica, Taipei, 11571 Taiwan; 2 Institute of Cellular and Organismic Biology, Academia Sinica, No. 128, Academia Road, Section 2, Taipei, 11529 Taiwan; 3 Institute of Chemistry, Academia Sinica, No. 128, Academia Road, Section 2, Taipei, 11529 Taiwan

**Keywords:** mRNA Technology, mRNA therapeutics, Lipid nanoparticles, Targeting mRNA delivery systems, New modality therapeutics

## Abstract

Realizing the immense clinical potential of mRNA-based drugs will require continued development of methods to safely deliver the bioactive agents with high efficiency and without triggering side effects. In this regard, lipid nanoparticles have been successfully utilized to improve mRNA delivery and protect the cargo from extracellular degradation. Encapsulation in lipid nanoparticles was an essential factor in the successful clinical application of mRNA vaccines, which conclusively demonstrated the technology's potential to yield approved medicines. In this review, we begin by describing current advances in mRNA modifications, design of novel lipids and development of lipid nanoparticle components for mRNA-based drugs. Then, we summarize key points pertaining to preclinical and clinical development of mRNA therapeutics. Finally, we cover topics related to targeted delivery systems, including endosomal escape and targeting of immune cells, tumors and organs for use with mRNA vaccines and new treatment modalities for human diseases.

## Introduction

Intensive studies on the therapeutic potential of messenger RNAs (mRNAs) for infectious disease and cancer have been ongoing for nearly three decades. A major obstacle in the development of this technology has been that delivery of naked modified mRNA is inefficient and results in low levels of protein production. To address this challenge, a variety of different delivery strategies have been evaluated. After decades of research and development, the first RNA-based therapy, patisiran (Onpattro™), reached the market in 2018 [1]. This lipid nanoparticle (LNP)-encapsulated small interfering RNA (siRNA) was approved by the United States Food and Drug Administration (FDA) for the treatment of hereditary ATTR amyloidosis, marking a significant milestone in the field and opening the door for mRNA-based drugs to be used in many applications.

mRNA technology provides a means of treating a broad array of different diseases. Major interest in the technology was stimulated by the speedy regulatory approval of the first mRNA vaccines, and many other mRNA vaccines or drugs are presently under evaluation in clinical trials [2, 3]. The first mRNA vaccines were rapidly developed to meet the worldwide need for prevention of COVID-19. Two mRNA-LNP vaccines against COVID-19, developed by Pfizer-BioNTech and Moderna, received US FDA approval in 2021 and 2022, respectively [4, 5]. Additionally, a new mRNA-LNP RSV vaccine was approved in May 2024 [6], highlighting the potential of mRNA technology in combating epidemics and pandemics. mRNA technology is ideal for this application, as it overcomes critical problems associated with conventional vaccines, such as complexity of manufacturing and the time needed for scale-up [7]. Moreover, new mRNA vaccines can be quickly created against different targets by simply changing the mRNA sequence.

Despite the short times required for development of new mRNA products, the initial journey from benchtop to clinical translation of mRNA took over a decade [2], and major efforts were required to develop stable mRNAs that could be translated in vivo [8–10]. Due to the high cost of mRNA production, its poor stability, and its high immunogenicity, pharmaceutical companies were historically less than enthusiastic about dedicating resources to extensive research and clinical trials on mRNA products. However, new strategies for mRNA modification, purification and sequence design revived interest in mRNA as a therapeutic modality [11].

In 1978, two different research groups demonstrated that mRNA could be successfully delivered into mouse cells and human cells by encapsulating the nucleic acids in liposome vesicles [12, 13]. However, the lack of a suitable delivery vehicle remained a critical challenge in the field for many years. It was widely accepted that delivery of naked mRNA shows low efficacy, so CureVac prepared a Protamine-mRNA complex that exhibits far better translation efficiency than naked mRNA [14]. The next major advance in mRNA delivery was the design of LNP-encapsulated mRNAs (mRNA-LNPs) that are not subject to the limitations of cationic liposomes or polymers. The inclusion of ionizable lipids in LNPs allows for efficient encapsulation of mRNA at neutral pH and endosomal escape at lower pH. After the LNP is taken up into the cell by endocytosis, ionizable lipids destabilize the endosomal membrane and release the encapsulated mRNA into the cytosol. The protection of mRNA cargo from degradation before cellular uptake and efficient release at the target site afforded by LNP encapsulation was key to the development of mRNA vaccines [15].

Currently, all FDA-approved LNPs are composed of four types of lipids: ionizable lipids, phospholipids, cholesterol and PEG lipids [16]. Among these components, PEG lipids are of concern because they may induce production of anti-PEG antibodies. Upon repeated injection of PEG-containing mRNA-LNPs, the anti-PEG antibodies will target PEG-coated mRNA-LNPs and reduce the delivery efficiency [17]. Although there have not been major safety concerns raised about mRNA vaccines, it is important to keep in mind that their clinical use is still relatively new, and the side effects and other limitations of mRNA medicines still need to be thoroughly studied.

Due to the broad potential for application of mRNA medicines and ease of manufacturing, many clinical trials have been initiated to evaluate mRNA drugs and vaccines. One major focus of current trials is cancer treatment and prevention, as mRNA drugs and vaccines are widely expected to be viable alternative treatments for cancers [18]. In general, cancer vaccines are designed to target tumor-associated or tumor-specific antigens (TAAs or TSAs). A vaccine with an mRNA sequence encoding a TSA or TAA can direct the immune system to recognize the antigen and thereby prevent cancer spread by killing the antigen-expressing tumor cells [19]. Sahin’s group first introduced the concept of individualized vaccines by implementing an RNA-based poly-neo-epitope approach to activate immunity against a variety of cancer mutations [20]. In addition to mRNA vaccines against TAAs/TSAs, mRNAs can be utilized as therapeutic agents. To enhance the therapeutic index of mRNA drugs and reduce potential side effects, researchers have generated strategies for targeting mRNA-LNPs to certain tissues or cell types. Targeting ligands may include antibodies, antibody fragments, peptides, aptamers, glycans or small molecules on the surface of the LNP that serve to enhance delivery of the target mRNA sequence to the disease site [21–24]. Successful targeting of mRNA-LNPs to tumors has been achieved via post-insertion or click chemistry methods, or by the inclusion of pH-sensitive lipids [25].

**Fig. 1:**
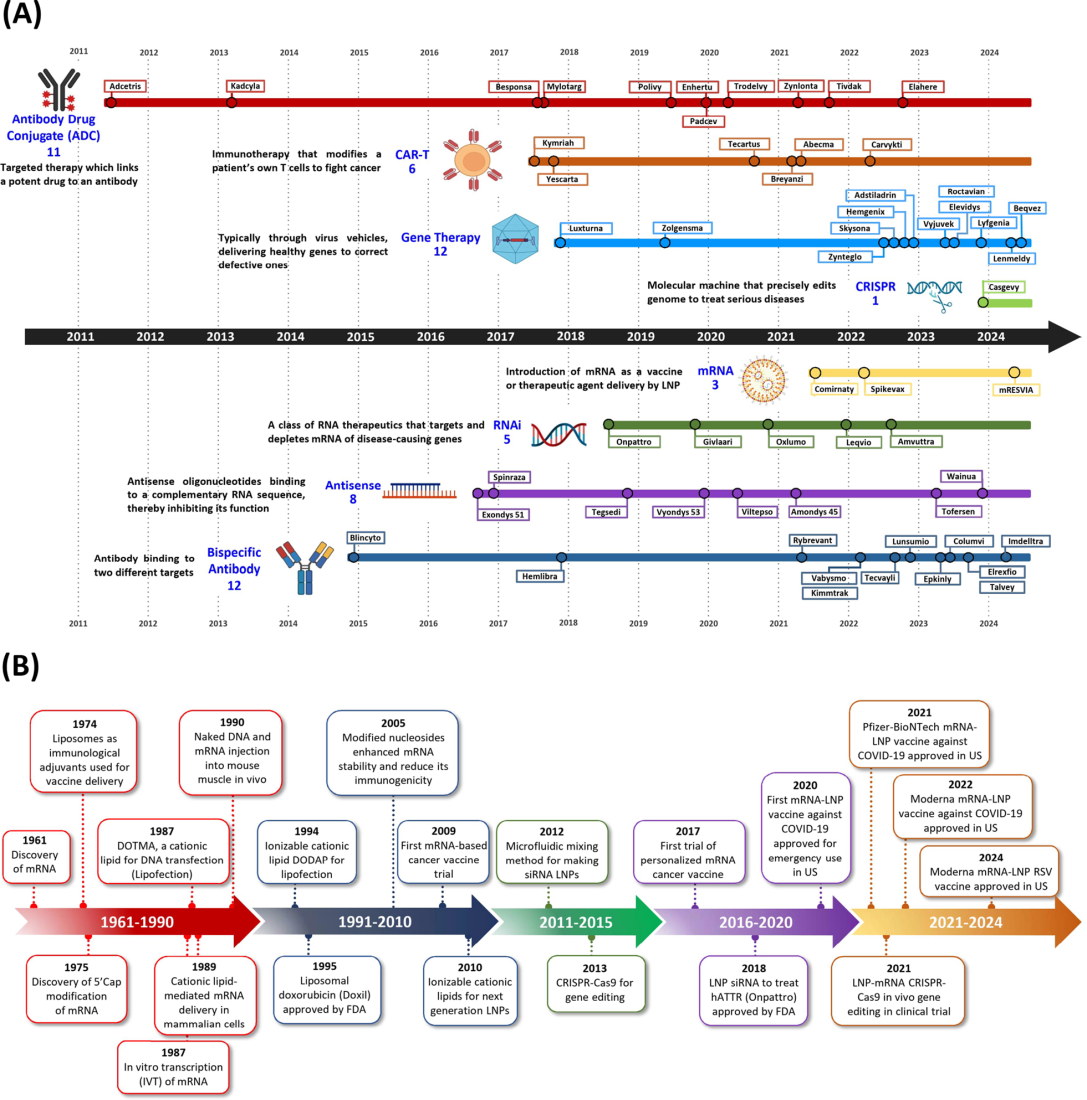
**A** Overview of new US FDA-approved therapeutic modalities. Recent technological breakthroughs have led to the introduction of several new therapeutic modalities and are expected to drive further innovation in the biopharmaceutical industry over the coming decades. The schematic illustrates a spectrum of new pharmaceutical modalities encompassing eight distinct categories: Antibody–Drug Conjugates (ADCs), gene therapy, chimeric antigen receptor T cell (CAR T) therapy, CRISPR-based therapeutics, messenger RNA (mRNA) therapeutics, small interfering RNA (RNAi), antisense oligonucleotides (ASOs), and bispecific antibodies. Prominent examples of pioneering US FDA-approved drugs within each modality are listed as follows. ADCs: Adcetris (brentuximab vedotin, approved 2011), bispecific antibodies: Blincyto (blinatumomab, approved 2014), RNAi: ONPATTRO (patisiran), ASOs: Exondys 51 (eteplirsen, approved 2016), CAR T therapy: Kymriah (tisagenlecleucel, approved 2017), Gene therapy: Luxturna (voretigene neparvovec-rzyl, approved 2017), mRNA therapeutics: Pfizer-BioNTech's Comirnaty (COVID-19 Vaccine, BNT162b2, approved 2021). Furthermore, the recent approval of a CRISPR/Cas9 gene editing therapy, Casgevy (exagamglogene autotemcel, approved December 8, 2023), underscores the continual expansion of therapeutic modalities. Numerous new cutting-edge technologies including mRNA technologies are currently under evaluation at various stages of drug development. **B** The graphic outline of milestones and development timeline in mRNA technologies and LNP delivery systems

Along with the introduction of mRNA drugs in recent years, major advances have also been made in the clinical implementation of several other modern therapeutic approaches, such as antibody–drug conjugates, bispecific antibodies and CRISPR technology. For example, Casgevy recently became the first FDA-approved CRISPR-based gene editing technology for the treatment of Sickle Cell Disease [26]. Additionally, CRISPR-Cas9 is being evaluated for in vivo delivery using mRNA-LNP system, with treating transthyretin amyloidosis initiated in 2021 [27]. In Fig. 1A, mRNA technology is contextualized among other innovative therapeutic modalities for design of drugs to treat various diseases. We further highlight several key breakthroughs in mRNA technology for vaccine and drug development in Fig. 1B, spanning from the discovery of mRNA in 1961 [28] to the US FDA approval of the RSV mRNA-LNP vaccine in 2024 [6, 29].

Recently, we published a comprehensive review of mRNA-based vaccines and therapeutics [2], providing an overview of the structural elements and chemical modifications of mRNA, various delivery systems, administration routes, and potential clinical applications of these therapeutics. The previous review article not only covered the basic principles of mRNA technology but also offered a detailed summary of RNA-based drugs that have reached clinical trials and received FDA approval.

In the current review, we provide an update on the advances in mRNA-based drug design and address ongoing challenges, such as storage and cold chain management of mRNA-LNPs as well as enhancement of endosome escape. In particular, we summarize the literature on ionizable lipid design and examine how different lipid structures impact the clinical effectiveness of mRNA-LNPs. We highlight recent innovations in lipid formulations that have shown promise in enhancing the delivery and performance of mRNA drugs. Furthermore, we describe recent progress in the development of mRNA drugs for cancer treatment. Our focus in this part is on approaches that have led to clinical trials, including immunotherapies and targeting of cancer-specific pathways. Additionally, we explore advanced strategies for functionalizing mRNA-LNPs with targeting ligands, such as glycans, peptides and antibody fragments, to achieve cell-specific or tissue-specific delivery. This review also looks ahead to forecast upcoming advances in mRNA-LNP technology and anticipates the next generation of mRNA drugs that could transform personalized medicine and cancer therapy.

## Synthesis and modification of mRNAs for use in vaccines and drugs

Messenger RNAs are the functional components of mRNA vaccines and drugs. In this section, we describe the molecular modifications, synthesis techniques and purification processes currently utilized in the development of mRNA molecules as pharmaceutical products. We also describe essential strategies for cold chain storage and transportation of mRNA-based medicines.

### mRNA modifications

A complete mRNA structure consists of several components, including a 5’ cap, 5’ untranslated region (UTR), coding sequence (CDS), 3’ UTR, and poly(A) tail. Capping is an essential process for creating functional mRNAs, as it modifies the ability of an mRNA to undergo processing and translation. Four main endogenous cap structures are known: cap0, cap1, cap2, and m6Am cap. In the cell, cap2-containing mRNAs account for about 50% of all mRNAs. Meanwhile, the m6Am cap is formed by N6 methylation and is found on about 30–40% of mRNAs [30]. While the molecular function of cap2 is not yet clear, it is known that the m6Am cap contributes to increase mRNA stability in cells [31].

For mRNAs produced by in vitro transcription (IVT), two major methods are used for capping the molecules. First, a cap analog called ARCA (anti-reverse cap analog) may be added by replacing the 3’ hydroxyl group of m7G with a methoxy group. ARCA-capped mRNAs generally have relatively high translation efficiencies and long half-lives [32–34]. The other major capping option is the co-transcriptional trimeric cap analog, which was successfully applied in SARS-CoV-2 mRNA vaccines [35–37].

To reduce immunogenicity of the mRNA, modified bases have been utilized in mRNA production. For instance, 5-methylcytidine (m5C), pseudouridine (Ψ) and N1-methyl pseudouridine (m1Ψ) have all been used for this purpose [38, 39]. Among these modifications, m1Ψ-containing mRNAs were shown to induce more protein production than m5C- and Ψ-containing mRNAs [40–42]. Although m1Ψ-containing mRNAs showed better protein production and stability, one study found that inclusion of unmodified uridine in an mRNA vaccine induces type I interferon-I (IFN-I) and its downstream signaling cascade to exert robust anti-tumor activity [43]. Another group further showed that innate IFN-I induction is not only stimulated by unmodified uridine, but it can also be promoted by ionizable lipid components like MC3 or KC2-LNP, but not L319-LNP [44].

Other characteristics of the mRNA have also been shown to affect protein production. For instance, codon usage is important consideration during mRNA design, as synonymous codons can contribute to different levels of protein production or affect protein folding and function [45–48]. In addition, more upstream open reading frames within an mRNA might titrate the translation initiation complex and affect protein translation [49, 50]. Several 5’ UTRs and the 3’ UTR from genes incorporated into mRNA templates were shown to improve expression and contribute to mRNA stability [51, 52]. In addition to the effects of the 5’ UTR and 3’ UTR, higher order secondary structures in the coding sequence region may also positively modulate mRNA functional half-life [53]. During mRNA-based drug development, all of these characteristics should be optimized to obtain the most suitable mRNA template.

### mRNA types

**Fig. 2:**
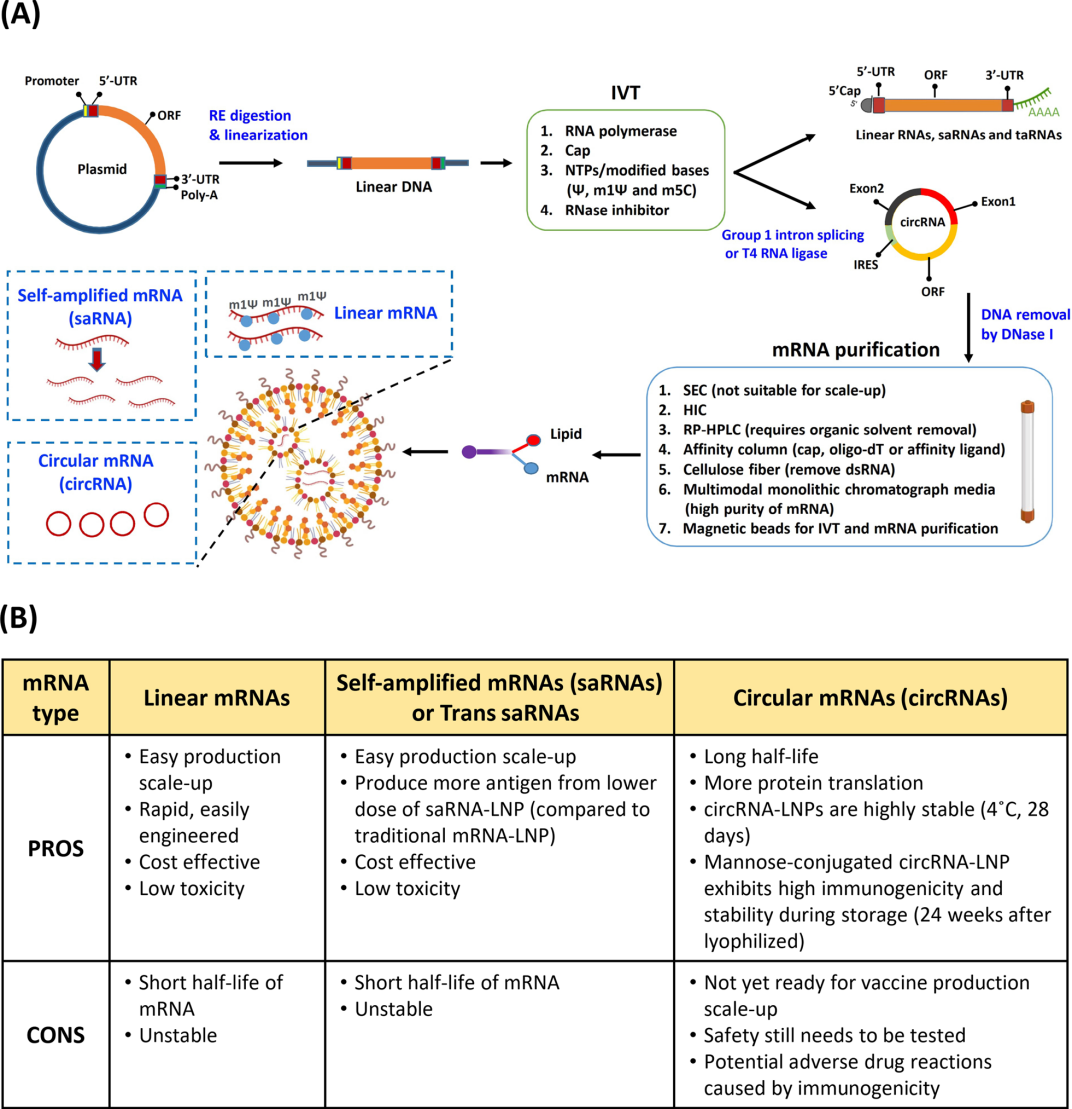
Synthesis and purification of three distinct mRNA types for mRNA-LNP drugs. **A** The in vitro transcription (IVT) process is illustrated. First, a plasmid is generated containing the target gene with a T7 promoter. After restriction enzyme (RE) digestion of the plasmid and purification of linear DNA, IVT is performed with T7 RNA polymerase, cap analogue, modified bases, and RNase inhibitor to generate transcribed linear mRNAs. The linear mRNAs may be traditional linear mRNAs, self-amplified mRNAs (saRNAs), or trans-amplified mRNAs (taRNAs). For production of circular RNAs (circRNAs), cyclization is achieved via intron-splicing reaction or T4 RNA ligase. Impurities within the mRNA products may be eliminated by DNA digestion and mRNA purification, along with other methods specific to the type of RNA product. The highly purified mRNAs are suitable for incorporation into mRNA-LNP formulations. (SEC: size exclusion chromatography, HIC: hydrophobic interaction chromatography, RP-HPLC: reverse phase HPLC). **B** Advantages and disadvantages of the three different mRNA types used for mRNA-LNP drugs are shown

In recent years, three different types of mRNAs have been applied in the development of mRNA drugs, including non-replicating mRNAs (nrRNAs), self-amplifying mRNAs (saRNAs) and circular mRNAs (circRNAs) (Fig. 2). A conventional mRNA is a linear nrRNA, which may contain many modifications to improve stability and expression (as described in 2.1). In contrast, saRNAs are derived from the positive-sense alphavirus genome and are composed of two regions. One region encodes the np1-np4 proteins that constitute a replication complex. The other contains amplification targets that encode capsid and envelope proteins (E3-E2-6K-E1), which may be replaced with an mRNA template of interest [54]. A major advantage of this technology is that small amounts of saRNAs are needed for injection. For instance, only 10 ng of saRNA can induce immunogenicity in mice, and only 5 μg of saRNA is sufficient for clinical testing [55, 56]. For its formulation, the saRNA construct can be divided into two segments in order to reduce the mRNA length and improve the encapsulation efficiency into LNPs. One mRNA construct would contain the alphavirus replicase transcript, and the other would carry the trans-replicon (TR)-RNA encoding a gene of interest with a subgenomic promoter to drive its replication. Using this approach, researchers have demonstrated that the TR-RNA of a bivalent vaccine against 2 viruses, chikungunya virus (CHIKV) and Ross River virus (RRV) can be amplified by trans-replication without loss of encapsulation efficiency in LNPs; most importantly, the researchers further showed that the treatment robustly induced immunogenicity toward the target [57].

Compared with linear RNAs, circRNAs are more resistant to exonuclease degradation due to their lack of 5’ and 3’ ends [58, 59]. This type of RNA is endogenously produced by noncanonical RNA splicing events, and some endogenous circRNAs are known to function as sponges for miRNAs or templates for stress-responsive peptides in mammalian cells [60]. For IVT-produced circRNAs, half self-spicing introns have been incorporated into a construct in order to induce splicing of an exon-linked target gene into a circular form; expression was driven by an internal ribosome entry site (IRES) in front of the target gene. Alternatively, linear IVT mRNAs may be ligated with T4 RNA ligase to generate a circular form [61–63]. An LNP-encapsulated circRNA has been tested as a SARS-CoV-2 vaccine and achieved better immunogenicity than a conventional LNP-encapsulated linear mRNA vaccine [64]. Additionally, a circRNA-encoded rabies virus glycoprotein encapsulated with a mannose-PEG forming LNP could be specifically delivered to dendritic cells and expressed antigen in lymph nodes to induce good immunogenicity. This vaccine could be kept at 4 °C for 24 weeks after lyophilization. Over this period, the vaccine targeting ability and immunogenicity were retained, demonstrating that LNP-encapsulated circRNAs may exhibit good stability without strict storage conditions [65]. The use of different RNA types in mRNA-LNPs is illustrated in Fig. 2a.

Improvements to mRNA stability and efficiency can be made by several different approaches, including base modifications or the use of saRNAs and circRNAs. While the use of both saRNAs and circRNAs seems to be growing, both types of RNA have key disadvantages (Fig. 2b). For example, saRNAs are limited by a potential safety concern that the alphavirus element may induce unwanted immune response; this concern will require careful attention in clinical studies. Although circRNAs show excellent inherent stability, which can support a longer mRNA half-life and more sustained protein expression, a major limitation of this approach is the complex manufacturing process. Besides selecting the most desirable characteristics of the mRNA, development of high efficiency, low toxicity mRNA vaccines and drugs also require optimization of a production process that can reliably generate pure mRNAs.

### mRNA purification and quality control

After synthesis by IVT, an mRNA product may contain many impurities, which can promote mRNA degradation. As such, different regulatory agencies have generated quality guides for mRNA vaccines. For example, the US Pharmacopeia (USP) released the “Analytical Procedures of mRNA Vaccine Quality” for Quality by Design (QbD) of mRNA manufacturing standards and analytic methods in April 2023. Removing impurities is a critical step for mRNA drug development, and mRNA has many physicochemical properties that can be utilized for purification. For instance, mRNA is a very large molecule with molecular weight often exceeding 300 kDa and a physical size more than 50 nm. These features make the molecule amenable to purification by size exclusion chromatography (SEC). Previously, mRNAs produced by IVT were separated from the DNA template, enzymes and excess nucleoside triphosphates (NTPs) using a Superdex-75 column or other SEC columns [66, 67]. Notably, the RNA conformation will affect SEC resolution, and double-stranded RNA (dsRNA) byproducts may not be separable due to their similarity in size to the single-stranded mRNA (ssRNA). Additionally, SEC is often not appropriate for scale-up in large-scale manufacturing. Another property that can be exploited for mRNA purification is the high hydrophobicity of the molecule [68]. As such, hydrophobic interaction chromatography (HIC) with suitable binding salts has proven to be an effective means of separating mRNAs from proteins, dsRNAs and short RNAs. A commonly used separation method in mRNA vaccine production is reverse-phase HPLC (RP-HPLC). Several studies have shown RP-HPLC purification can eliminate dsRNA-induced immunity and increase translatability by 10- to 1000-fold compared to non-HPLC-purified mRNAs [69–71]. However, HPLC still has limitations, such as the potential use of toxic organic solvents.

Another approach for mRNA purification is affinity columns, such as oligo-dT columns that can effectively remove impurities without poly(A) tails. This method has been applied for SARS-CoV-2 and anti-influenza immunoglobulin G (IgG) mRNAs, but it still cannot provide good separation of ssRNA and dsRNA [72–74]. One study showed that a Cap affinity column was more efficient for mRNA purification than an oligo-dT column [75]. In the case of circRNAs, the constructs may be efficiently purified with HPLC [64, 76, 77]; however, HPLC is not usually amenable to scale-up. An alternative strategy to remove precursor and intron RNAs is affinity purification with highly selective affinity ligands [78]. Since dsRNA is very difficult to remove by separation technologies, enzyme digestion with RNAIII may be needed, as this enzyme can digest dsRNA without affecting mRNA integrity [79]. Alternatively, dsRNAs may be removed by cellulose fibers in an ethanol-containing buffer due to specific binding by 2-hydroxyl residues in the dsRNA. This method is scalable and was shown to achieve 90% removal of dsRNA with more than 65% mRNA recovery [80]. One recent report introduced a highly efficient chromatographic method with properties of ionic exchange and hydrogen bond force adjustment, called multimodal monolithic chromatography media (CIM PrimaS); this method could be used to generate high purity mRNA at pH 10.5 [81]. Currently, IVT is performed with magnetic beads conjugated with target gene PCR product, and purification of IVT mRNAs is accomplished using of oligo-dT-conjugated magnetic beads. This method offers a straightforward and expeditious approach to mRNA purification [82]. RNA purification strategies are detailed in Fig. 2a.

While impurities must be removed from IVT-generated mRNA, it is possible that chromatographic purification will affect mRNA structure and biological function. Therefore, further development of high-efficiency purification technologies is an ongoing pursuit. In this regard, utilization of specific ligands for combination or sequential purification strategies might offer a new pathway to improve the purity and yield of IVT-generated mRNAs [83].

### Storage and cold chain management

The storage conditions of drugs have a significant impact on their effectiveness. Improper storage can lead to a loss of potency, reduced therapeutic efficacy and increased safety risks. Generally, the small molecules and biological products most often seen in clinical trials and on the market are stored at either room temperature, 4 °C or − 20 °C. In contrast, the FDA-approved mRNA vaccines BNT162b2 (Comirnaty®) and mRNA-1273 (Spikevax®) require storage at low or ultra-low temperatures (− 80 °C); despite this unusual requirement, the vaccines were successfully deployed and able to make meaningful impacts on the COVID-19 pandemic [84, 85]. Nevertheless, the requirement for cold chain transport and storage of these vaccines substantially hindered their clinical application and dissemination, largely due to a lack of transport links, refrigeration facilities or stable power supplies, especially in third-world countries.

The instability of mRNA-LNPs during storage is primarily attributable to chemical degradation by hydrolysis and oxidation reactions. Hydrolysis can lead to the cleavage of phosphodiester bonds in the mRNA backbone, while oxidation may result in base cleavage and alterations to the mRNA secondary structure [86, 87]. Therefore, mRNAs may be quickly degraded if stored for prolonged periods in an aqueous environment. In recent years, studies have been performed to evaluate whether freeze-drying methodologies can be used to augment stability, extend the shelf life, and broaden the storage temperature range of mRNA-LNP products. In a phase III clinical trial, lyophilized mRNA lipid nanoparticles (mRNA-1647) were tested for their ability to protect against cytomegalovirus (CMV) infection [88] (ClinicalTrials.gov: NCT05085366). It was observed that product storage at 5 °C ensured a shelf-life of at least 18 months [89]. Several additional studies have corroborated the improved stability of lyophilized mRNA lipid nanoparticles. For instance, Ai et al. showed that long-term (6 months) storage of lyophilized mRNA-LNPs at 4 °C and 25 °C did not lead to any measurable changes in physical size, polydispersity index (PDI), encapsulation efficiency (EE), mRNA integrity or lipid degradation. Moreover, the lyophilized mRNA-LNP against the omicron variant retained high immunogenicity similar to freshly prepared omicron mRNA-LNP even after storage at 4 °C or 25 °C for 6 months [90].

**Fig. 3:**
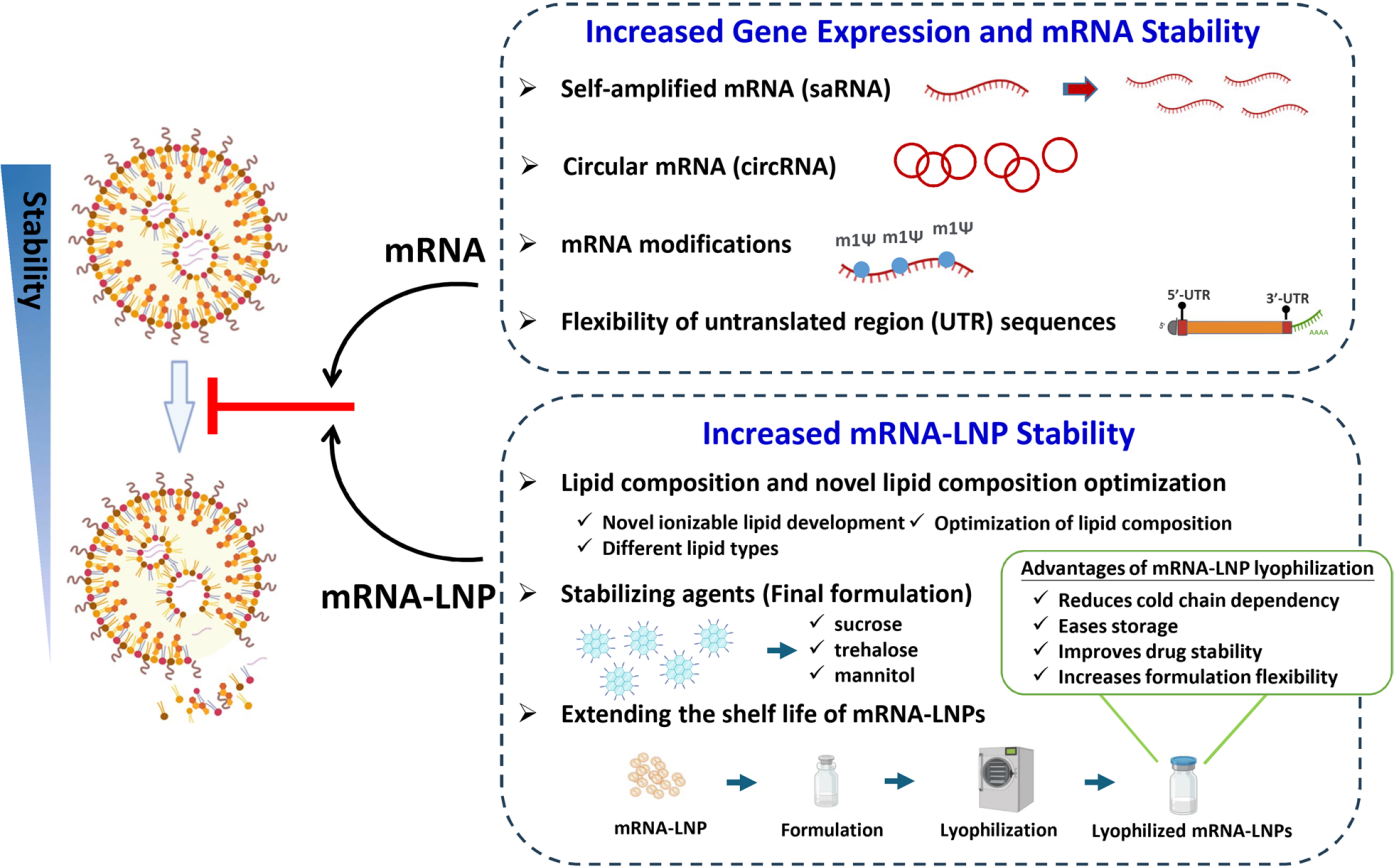
Strategies for enhancing the efficacy and stability of mRNAs and mRNA-LNPs. Diverse strategies may be used to preserve the integrity of mRNAs and mRNA-LNPs. Different characteristics of the mRNA can augment stability and prolong intracellular expression of the encoded product. Such characteristics include self-amplifying or circular mRNA, nucleotide sequences, and untranslated regions (UTRs). Furthermore, optimizing lipid formulations through adjustment of lipid ratios or inclusion of novel components can improve the stability of mRNA-LNPs. Recent studies indicate that lyophilization in the presence of appropriate cryoprotectants can facilitate the long-term storage of mRNA-LNPs at 4 ℃, which would be a major advantage for future clinical applications

During the lyophilization process, cryoprotective reagents are critical for preventing mechanical disruption of the mRNA-LNPs due to ice crystals. The most common cryoprotectants encountered in the literature for freeze-drying microparticles are sugars, such as trehalose, sucrose, glucose and mannitol [91, 92]. Pfizer-BioNTech and Moderna Covid-19 mRNA-LNP formulations both include sucrose as a cryoprotectant to maintain LNP integrity during freezing [93]. However, not all sugars can serve as effective cryoprotectants for mRNA-LNPs. For instance, the crystallization of 20% mannitol during freezing and monosaccharides like fructose and glucose may lower the glass transition temperature and lead to collapse of the nanoparticle [94]. In addition, Li et al. used scanning electron microscopy and transmission electron microscopy to observe structural changes in mRNA-LNPs after freeze-drying. They found that a mixture of sucrose (8.8%), trehalose (2%), and mannitol (0.04%) in the freeze-drying solution caused the mRNA-LNPs to exhibit a ginger root-shaped rigid structure with large porosity. This structure could rapidly adapt to temperature changes and efficiently exclude water molecules, reducing the lyophilization time [95]. Overall, these findings suggest that lyophilization of mRNA-LNP might help to overcome instability, improve stability, and eliminate the necessity of cold chain transport and storage (Fig. 3).

Notably, different compositions and ratios of LNP components can greatly affect the physical and chemical properties of an mRNA-LNP product. One study evaluated the effects of five ionizable lipids on stability after the LNPs were stored at temperatures of 2–8 °C, 25 °C, and 40 °C for at least 9 weeks. Among the tested ionizable lipids, C12-200 showed 90% higher eGFP protein expression in HEK293 cells compared to the others after 11 weeks of storage at room temperature [96]. Further investigations into structure–activity relationships and Design of Experiments (DoE)-informed studies will be needed to better understand how the stability of LNPs may be affected by current and new ionizable lipid candidates.

## Design and development of novel lipids for LNP delivery systems

In recent years, LNPs have emerged as a promising vehicle for delivery of nucleic acid therapeutics, owing to their ability to shield cargoes against degradation and facilitate cellular uptake [97]. Typically, LNPs consist of four essential lipid components: (i) cationic/ionizable lipids, (ii) helper phospholipids, (iii) PEGylated lipids, and (iv) cholesterol [97, 98]. Ionizable lipids exhibit pH-responsive behavior, interacting with mRNA at neutral pH to provide stability and shield against degradation, while also facilitating endosomal escape by destabilizing membranes in acidic environments [98, 99]. Helper phospholipids enhance nanoparticle stability, rigidity and biodistribution, ultimately improving transfection efficiency and endosomal escape [99]. PEGylated lipids extend systemic circulation times and reduce immune recognition by influencing particle size, uptake efficiency, and target specificity [97, 99]. Lastly, cholesterol contributes to the biocompatibility and structural integrity of the nanoparticle, potentially enhancing transfection efficiency and endosomal escape [98]. In this section, we summarize current knowledge regarding the design and development of these key lipid components, with an emphasis on advances made over the past three years.

### Ionizable/cationic lipids

#### Design and development of ionizable and cationic lipids

It is well established that the intracellular concentrations of glutathione (GSH) and other reductive species are orders of magnitude higher than those in the extracellular environment (GSH_cytoplasm_: GSH_extracellular_ > 1000: 1). This unique feature of the intracellular milieu can be utilized to facilitate the degradation of bioreducible mRNA-containing LNPs, leading to efficient mRNA release inside cells. Drawing on this principle, a panel of LNPs featuring multi-tail lipidoids with bioreducible disulfide bonds were compared in an in vivo FLuc mRNA delivery assay. In this study, **306-O12B** LNP was most effective at facilitating mRNA delivery to murine liver, as compared to analogous formulations and the MC3 LNP [100]. In a similar study, the **113-O12B** LNP exhibited a propensity for lymph node localization in mice following subcutaneous administration of encapsulated FLuc mRNA, despite its low overall delivery efficacy [101]. The results of lipid component screens suggest that delivery efficacy is influenced by alkyl chain length (C8 > C6 > C10 > C12) and the spacing between amine atoms (C3 > C2). Furthermore, decreasing the count of branched tails or substituting the central methyl amine with a piperazine nucleus, ethyl, hydroxyl or *N*-(1,2-ethanediyl)acetamide groups significantly decreases delivery efficiency. Another study demonstrated that substituting the ester linkage on the **306-O12B** with an amide bridge (**306-N16B**) causes LNPs to selectively deposit FLuc mRNA in pulmonary tissues [102]. Moreover, cellular populations within the lungs can be specifically targeted by tuning the amino head. Among compounds of its chemotype, **306-N16B** appears to be the most promising, despite the divergent finding that longer alkyl chains on the lipidoids (C12 > C10 > C8) are positively correlated with luminescence intensity in the in vivo FLuc mRNA delivery assay.

Mechanistic studies involving proteomic analyses further revealed that different distributions within the protein coronas significantly influence the target ability of LNPs. An ionizable lipid with a degradable linker (**4A3-SCC-PH**) and branched tails demonstrated superior performance in mediating mRNA transfection, exhibiting a remarkable 15.5-fold enhancement in FLuc mRNA delivery compared to the MC3 LNP [103]. The effect was attributed to the presence of asymmetric alkyl chains tethered on the thioether on **4A3-SCC-PH**, combined with GSH-responsive characteristics inherent in the disulfide bond, which give the molecule a conical geometry. These features were presumed to have facilitated the efficient delivery of mRNA cargo within the intracellular environment of malignant cells.

Structural optimization efforts have led to significant advancements in the delivery of biologics to T lymphocytes, addressing a key challenge of low transfection efficacy in this cell type. For example, the lipidoids **93-O17S** and **9322-O17S**, which feature imidazole-based structures with chalcogen (O, S, Se)-containing tails (Table 1), can mediate efficient delivery of reporter mRNA in T lymphocytes. Detailed structural screening revealed that the length and branching of the spacer between the amine head and the tail structures are highly correlated with delivery potency. Additionally, the presence of heteroatoms (O or S, or S–S) in the tail structures is crucial for effective mRNA delivery. Notably, **93-O17S** showed 8.2% and 6.5% delivery efficacy in CD4 + and CD8 + T lymphocytes, respectively [104].

**Table 1:**
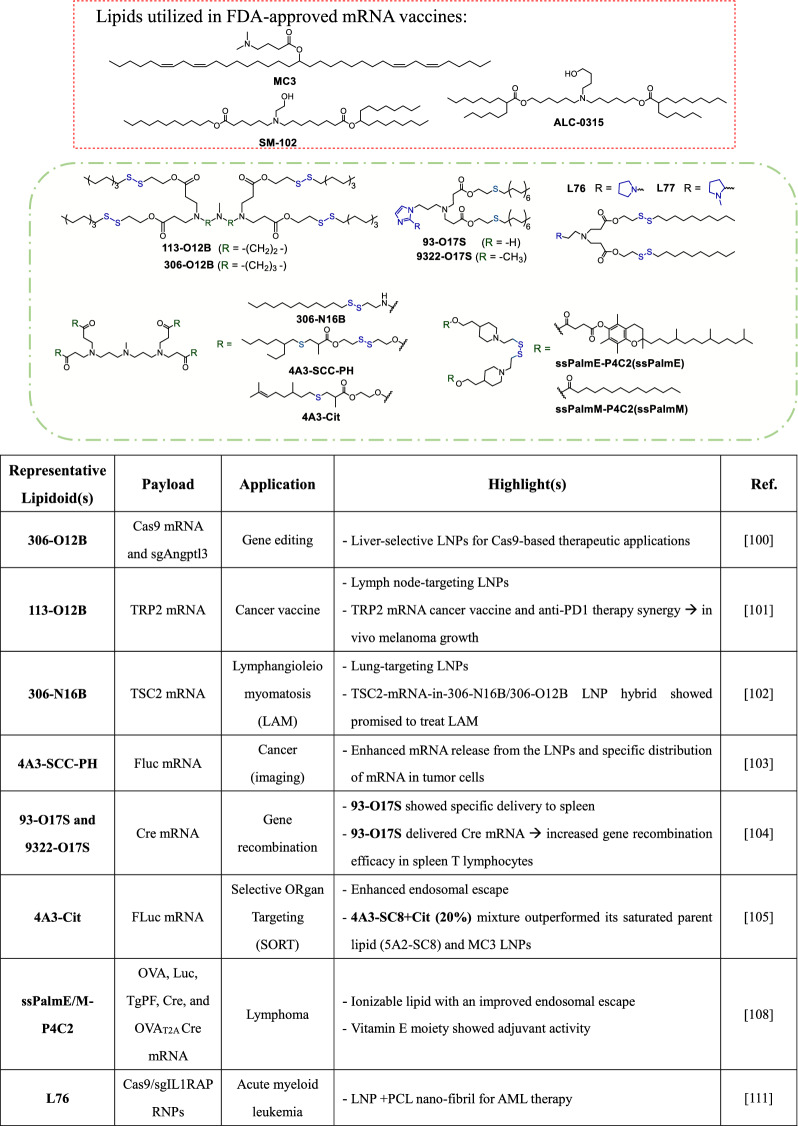
Selected ionizable lipids containing bioreducible disulfide bonds

Several studies have sought to optimize ionizable lipids in LNPs by modifying aspects such as the ionizable amine core, ester-based degradable linker, or the thioester tail periphery. However, it remains largely unknown how unsaturated bonds in the thioester tail structures affect delivery potency. To address this issue, Lee et al. synthesized alkenyl thiolesters and integrated the molecules into the tail structures of potential ionizable lipids. While the introduction of unsaturated bonds in the tail improved transfection for some lipids, a direct positive correlation between this property and enhanced delivery was not conclusively established. Among the screened series, **4A3-Cit** was associated with the highest luminescence expression (Table 1), outperforming **4A3-Ne**, a structural analogue that differs from **4A3-Cit** due to a prenyl motif in each tail. This result suggests that increased tail rigidity may influence delivery potency. Furthermore, the study showed that tail unsaturation did not significantly alter the biodistribution of LNPs, but incorporation of 20% **4A3-Cit** into the saturated parent lipid counterpart led to an 18-fold increase in average luminescence signal in the liver over the original formulation, highlighting the importance of tail unsaturation for mRNA delivery [105].

Akita’s group has developed a series of self-degradable ionizable lipids known as **ssPalmX** (X = M, O, L), which include an SS-cleavable bond and pH-activated lipid-like structure [106]. These lipid motifs have been shown to contribute to enhanced endosomal escape and mRNA release [107]. Recently, the **ssPalmE** lipid (with a vitamin E scaffold) was modified to **ssPalmE-P4C2** and used to encapsulate IVT-generated ovalbumin mRNA. This mRNA-LNP was found to induce specific cytotoxic T cell responses [108]. A comparison between empty **ssPalmE-P4C2** LNP and **ssPalmM-P4C2/ssPalmO-P4C2** LNPs showed that only the empty **ssPalmE-P4C2** LNP induced ovalbumin-specific cytotoxic T cell activity and increased the concentrations of cytokines/chemokines, including interleukin-6 (IL-6), keratinocyte-derived cytokine (KC), monocyte chemoattractant protein-1 (MCP1) and IFN-gamma-inducible protein 10 (IP-10), which suggests that **ssPalmE-P4C2** LNP possesses adjuvant activity.

Xu’s team has also contributed significantly to the development of ionizable and bioreducible lipids as nanocarriers for genome-editing proteins [109]. Specifically, this group showed that pyrrolidine-based LNPs combined with S- or Se-containing ether tails, exhibit high transfection efficiency and lower cytotoxicity than Lipofectamine 2000 [110]. In addition, the bioreducible lipids **L76** and **L77** were identified as the most promising for gene editing applications. These two lipids were used to encapsulate Cas9/single guide RNA (sgRNA) ribonucleoprotein targeting the *IL1RAP* gene, and the products showed superior gene editing efficiency in leukemia cells [111]. Notably, **L76** has a linker directly connected to position one of the pyrrolidine, and **L77** has a linker connected to position two of the pyrrolidine. Both lipidoids exhibited slightly lower cytotoxicity and conferred higher gene editing efficiency when coated on mesenchymal stem cell membrane-coated nanofiber (MSCM-NF).

In order to deliver siRNA to leukocytes, Peer's team developed various ionizable lipids resembling MC3 by employing different linker moieties, such as hydrazine, hydroxylamine and ethanolamine [112]. These lipids formed LNPs of distinct sizes, and the study results showed that linoleic acid chains contribute to higher efficacy than ester-based chains. Among all tested linkers, ethanolamine displayed the best performance. LNPs with a surface pKa of 5.5 to 7.0 effectively silenced genes in vivo, corresponding to the pKa of ethanolamine (range, 6.2 to 6.5). Notably, encapsulation of siPLK1 with the piperazine head group-containing **lipid 10** (Table 2) led to superior silencing effects at low siPLK1 concentrations, as compared to encapsulation with MC3 LNP. Biodistribution studies in mice further revealed that piperazine head group lipids exhibited high levels of spleen accumulation. Additionally, efforts to target T lymphocytes and using anti-integrin β7 monoclonal antibody were also successful, as evidenced by CD45 downregulation. In another study, Peer and his team introduced two novel ionizable lipids, including the piperazine-containing **lipid 2** and another acyclic lipid analogue for LNP-based SARS-CoV-2 vaccine applications [113]. Interestingly, LNP formulations with **lipid 2** induced a robust cellular immune response when administered intramuscularly, while formulations with the acyclic analogue expressed superior immunogenicity when administered intradermally [113]. To assess lipids in the context of transient RNAi for cancer treatment, researchers utilized **lipid 10**-modified LNPs with hyaluronic acid (HA) surface modifications to facilitate cancer cell internalization via CD44 receptor targeting [114]. Encapsulation of siPLK1 and sieIF3c in these LNPs led to effective gene suppression, with 50% cytotoxicity occurring at concentrations below 18 nM. In late-stage ovarian cancer in situ models, this treatment was associated with median survival time exceeding 80 days and a 60% survival rate. Similarly, another study demonstrated that siCKAP5-carrying LNPs could inhibit tumor growth and lead to an 80% increase in survival rate of mice implanted with NAR cells [115].

**Table 2:**
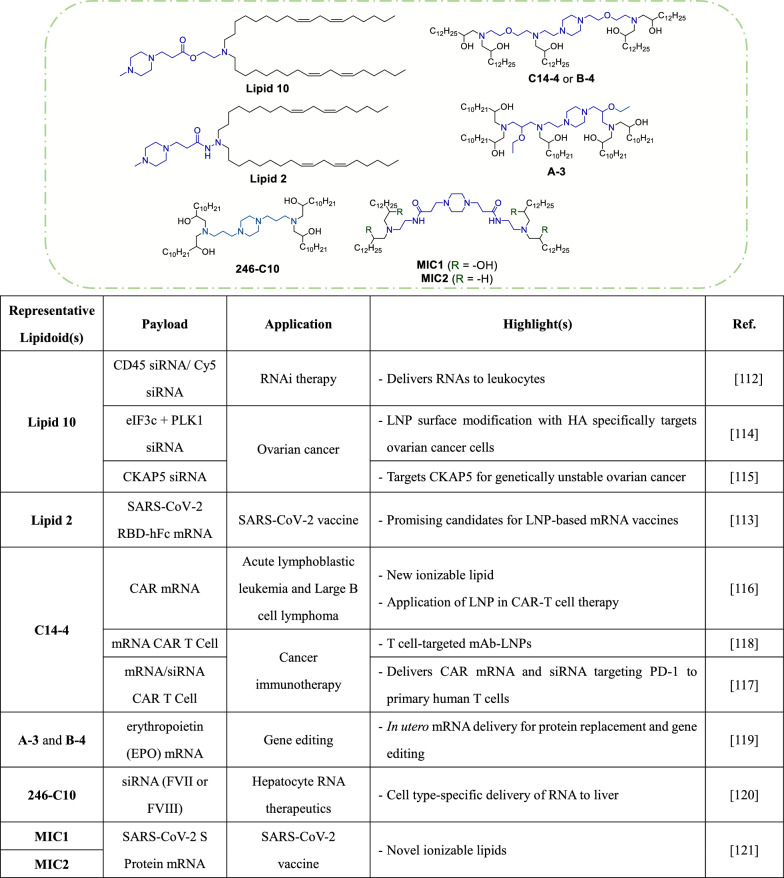
Selected ionizable lipids with cyclic tertiary amine scaffolds

For another set of studies, Mitchell's team synthesized 24 ionizable lipids to evaluate targeted T cell-specific mRNA delivery. The candidate molecules comprised polyamine cores centered on aminoethylpiperazine (AEP) and epoxy-terminated alkyl chains [116]. Evaluations in Jurkat cells revealed lower levels of mRNA delivery with shorter (C12) and longer (C16) alkyl chains, similar to lipofectamine. Meanwhile, moderate chain lengths (C14) with polyamine cores showed more favorable efficiency. Branches or cyclic connectors in the polyamine cores caused the LNPs to exhibit lower efficiency, whereas PEG or ether groups improved delivery, with ether groups yielding the best results (**Lipid C14–4**). In primary human T cells, the best formulation could be used to efficiently and persistently suppress PD-1 expression, indicating a long-lived PD-1 knockout effect [117]. In vivo experiments in mice revealed that **C14–4** LNPs preferentially accumulated in the spleen [118], whereas another study showed that LNP formulation with the same lipid designated as **B-4** exhibited a strong luciferase signal in the liver [117]. Moreover, **A-3** and **B-3** LNPs showed robust effects, likely due to their branched structures. The **A-3** LNP demonstrated superior mRNA delivery to the fetal liver, particularly when evaluated by GFP transfer. This result highlights the importance of the ethoxy group for activity. Additionally, tests with erythropoietin mRNA showed higher protein production levels in the fetal liver with delivery by **A-3** LNPs in the short term, while **B-4** LNPs led to higher levels in the long term. Minimal differences in survival were observed in C57BL/6 and Balb/c mouse strains treated with the various LNPs [119].

In a study by Lee’s team, FLuc mRNA delivery with the **246C10** LNP, which features a piperidine head and four hydroxyl groups (Table 2), led to a 400-fold increase in FLuc expression in serum-free culture medium. Furthermore, anti-LDLR antibodies were applied to impede **246C10** LNP cellular uptake, and a significant inhibitory effect was observed. Intravenous administration of **246C10** LNP to mice revealed that the highest luciferase expression could be observed in the liver. Finally, the authors found that adjusting the proportions of PEG-lipid did not effectively target liver sinusoidal endothelial cells (LSECs), but the addition of glucose-PEG resulted in efficient LSEC targeting [120].

Song's team developed a series of 4N4T ionizable lipids tailored for delivery of mRNA for the full-length S protein of SARS-CoV-2. The lipids are designated as "4N" to represent the four tertiary amine nitrogen atoms and "4 T" for the four hydrophobic tails. The lipids were evaluated in terms of antigen-presenting cell (APC) S protein expression levels, with **MIC1**/**MIC2** showing the best performance (Table 2). The superior efficacy of **MIC1**/**MIC2** may stem from their multi-charged nature and higher amine to phosphate ratio, which facilitates efficient endosomal escape of LNPs. In vitro experiments demonstrated excellent expression of the encapsulated mRNA, and in vivo experiments demonstrated excellent safety profiles [121].

The ether analogue **HEAH** (Table 3) was developed as a more stable alternative to ALC-0315 LNPs, as it showed enhanced stability after storage at 37 °C for 1 month. Moreover, **HEAH** LNPs exhibited a 2.77-fold higher delivery efficiency for FLuc mRNA in mice and had a safer toxicity profile than ALC-0315 LNPs. These findings suggest that modifying the ester bond and introducing a hydroxyl group may significantly impact the potency and stability of LNPs [122].

**Table 3:**
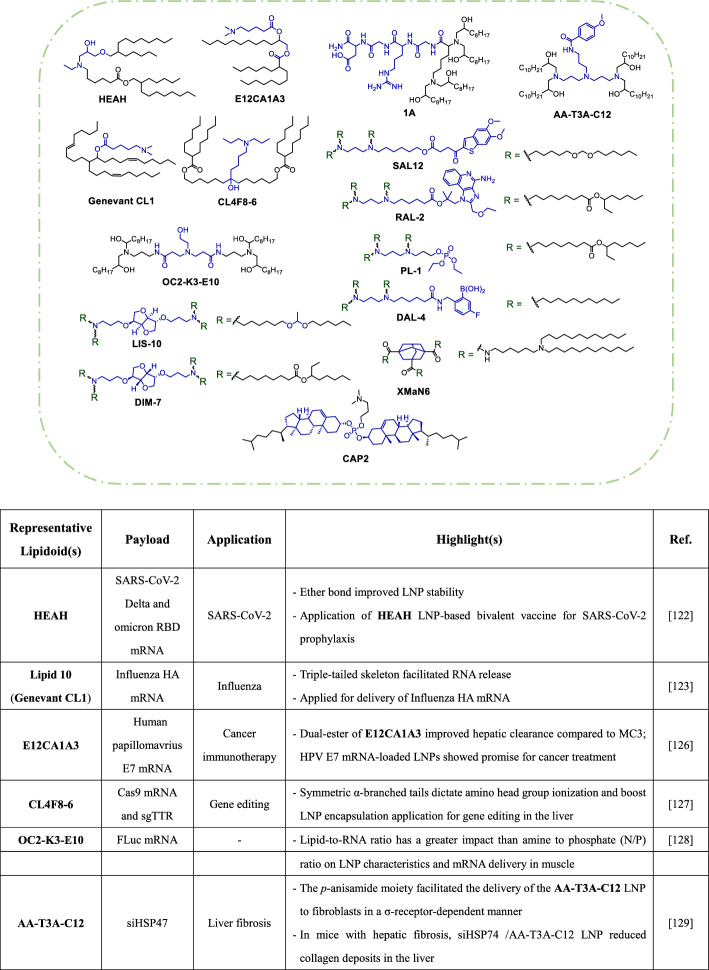

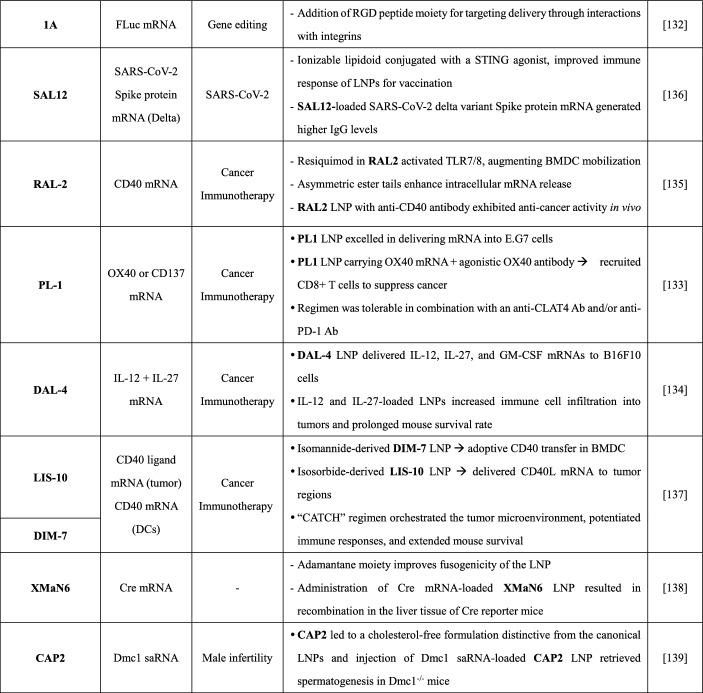
Selected ionizable lipids with acyclic tertiary amine scaffolds

A SAR study on **Genevant CL1** revealed that medium-length tails (C9 and C10) and increasing degrees of unsaturation in the *cis* configuration enhance its performance. The triple-tailed skeleton with additional branching points contributes to a cone-shaped molecular architecture that promotes release of the mRNA cargo. In murine hemagglutinin mRNA vaccine models, intramuscular administration of mRNA in **Genevant CL1** LNPs induced higher IgG titers than SM-102 LNPs, which had similar performance to MC3 and ALC-0315 LNPs. Of note, **Genevant CL1** is utilized in ChulaCov19, a non-stabilized prefusion COVID-19 mRNA vaccine currently in phase II trials [123–125].

In other studies, researchers aimed to improve the hepatic elimination of lipidoids by altering the positions of ester linkages to influence liver clearance [126]. Among hundreds of 1, 2-diesterified amino lipidoids tested in mLuc-loaded LNPs, **E12CA1A3** showed superior in vitro transfection efficiency, as compared to other lipidoids and MC3 (Table 3). Pharmacokinetic profiles in mice receiving ovalbumin mRNA in optimized **E12CA1A3** LNPs suggested higher clearance and hepatic extrusion of **E12CA1A3** compared to MC3, though no comparison was made with the diesterified analogue ALC-0315.

To understand the relationship between branched lipidoids, their physicochemical properties, and biological functions, a library was created of 32 lipidoids with various types of α-branched tails [127]. Comparative studies on this library showed that an appropriate length of each linear alkyl chain within the tails (C6–C12) enhanced the amino heads in the lipidoid structures, resulting in improved microviscosity and greater endosomal escape of LNPs. Symmetric lipidoids had distinct mRNA biodistributions; those with a total carbon number of C11–C14 in each tail accumulated preferentially in liver, while those with C14–C18 tails showed selective accumulation in the spleen. Among the screened molecules, **CL4F8–6** LNPs emerged as the most promising for carrying Cas9 mRNA and sgTTR. This combination resulted in 54% hepatic genome editing and a 77% reduction in circulating TTR protein levels after a single intravenous dose in mice.

A different lipidoid library with tri-ionizable amino heads was constructed using iterative design (Table 3). From this library, **OC2-K3-E10** showed comparable performance to SM-102 for LNP-mediated delivery of FLuc mRNA in mice [128]. SAR analysis revealed that a hydroxyl group near the core amine was crucial for mRNA expression, while those in the tails increased lipid-mRNA interactions with a minor impact on expression. Additionally, a three-carbon linker between the amide and central amine improved mRNA expression, and encapsulation efficiency was influenced by tail length.

An alternative approach to designing liver-targeting lipidoids involves incorporating a ligand with high affinity for liver-associated cells, as exemplified by **AA-T3A-C12** [129]. The **AA-T3A-C12** molecule is derived from *p*-anisamide, a ligand of σ-receptor expressed on activated fibroblasts [130]. When formulated in an LNP, **AA-T3A-C12** showed enhanced association with σ-receptor-mediated delivery of siGFP in activated 3T3-GFP fibroblasts (Table 3). The LNP delivery efficiency was significantly influenced by the number of amino groups present on the lipidoids to which *p*-anisamide was attached. In mice with CCl_4_-induced hepatic fibrosis, intervention with siHSP47-loaded **AA-T3A-C12** LNPs resulted in more efficient knockdown of HSP47 and reduced collagen deposits in the liver, as compared to hepatocyte-targeting MC3 LNPs [131]. Another lipidoid, **1A**, is derived from the RGD peptide, a specific ligand of α_V_β_3_ and α_5_β_1_ integrins that are overexpressed in some solid tumors. FLuc mRNA-LNPs with a **1A/C12-200** ratio of 0.2 induced twofold higher protein expression in HepG2 cells than the original **C12-100** LNP formulation, [132].

Dong's team has derived ionizable lipidoids from 1,3-propyldiamine by incorporating various ligands or small molecules [133–136]. Notably, **SAL12** (Table 3) is equipped with a non-nucleotide STING agonist, which can enhance the immune response to LNPs used in vaccines [136]. In vitro screening revealed that the acetal linkers in **SAL12** improve the delivery of FLuc mRNA to murine bone marrow-derived dendritic cells (BMDCs), as compared to its ester, carbonate and alkyl counterparts. Immunization of mice with Spike protein mRNA from the SARS-CoV-2 delta variant in **SAL12** LNPs generated fivefold higher IgG titers after 4 weeks and threefold higher titers after 10 weeks than ALC3-0315 LNPs, without causing apparent toxicity. Similarly, **RAL2** (Table 3) has an incorporated TLR7/8 agonist, resiquimod, which enhances dendritic cell activation [135]. Unlike **SAL12**, the asymmetric ester tails on **RAL2** contribute to a packing parameter of 2.3. This value suggests a tendency toward assembly of a reverse-hexagonal (HII-phase) architecture, which can facilitate intracellular mRNA release. The therapeutic potential of **RAL2** is evidenced by the prolonged survival of a mouse cancer model following systemic administration of therapeutic-carrying LNPs.

One direct approach to T cell activation is to upregulate co-stimulatory receptors with an agonist [133]. A series of biomimetic lipidoids was designed to exert this function based on a 1,3-propyldiamino core equipped with either phosphotriester heads or acetylated saccharide heads. Phosphotriester lipidoids with ester tails, such as the asymmetric **PL1** (Table 3), showed superior delivery of FLuc mRNA into E.G7 cells when formulated into LNPs. Notably, the length of alkyl chains played an important role in the activity of the glycolipidoids. Another means of activating T cells is the indirect strategy. For this strategy, pro-inflammatory cytokines within local tumor regions are utilized to recruit and activate T cells, thus avoiding potential systemic adverse effects [134]. A series of 1,3-propyldiamino lipidoids was prepared with diverse aryl fragments ligated by an amide bond. The series was then formulated into Luc mRNA-LNPs. In vitro screening revealed that substituents [e.g., F, OH and B(OH)_2_] on the phenyl moiety of lipidoids improved the encapsulation efficiency of LNPs. Nevertheless, only the **DAL-4** LNP was able to give rise to a significant luciferase signal in B16F10 cells (Table 3). Unlike the MC3 LNP, this system could be used to successfully transport interleukin-27 (IL-27), interleukin-21 (IL-21) and granulocyte–macrophage colony-stimulating factor (GM-CSF) mRNAs into B16F10 cells.

Concurrent targeting of tumor cells and immune cells represents a novel approach to cancer therapy. LNPs composed of tetra-tailed lipidoids with D-isomannide, D-isosorbide, or L-isosorbide scaffolds were screened for their ability to deliver FLuc mRNA to BMDCs [137]. Despite the absence of clear SARs, LNPs containing either **DIM-7** with a D-isomannide core and esters or those containing **LIS-10** with a L-isosorbide nucleus and acetal linkers showed superior performance (Table 3). The optimized **DIM-7** LNP had a delivery efficiency that ranged from 3- to 20-times greater than those of ALC-0315, MC3 and SM-102 LNPs. In the "CATCH" regimen, **DIM-7** LNPs were used for adoptive CD40 transfer in BMDCs ex vivo. The **LIS-10** LNPs were applied to deliver CD40 ligand mRNA into tumor regions in order to initiate immunogenic cell death.

One versatile lipid-like material for LNPs is **XMaN6** (Table 3), which may be well suited for use in various applications [138]. The adamantane-based structure of **XMaN6** confers a tripodal geometry that improves LNP fusogenicity. This property supports high encapsulation efficiencies (80–90%) and robust in vitro delivery activities for mRNA, siRNA and pDNA cargoes. Of note, in vivo experiments showed no apparent signs of **XMaN6** toxicity. Moreover, lipidoids are not only limited to molecules with long-chain fatty acids and alkyl groups. For instance, cholesterol-amino-phosphate (CAP) analogs mimic biological membranes by integrating phospholipids and cholesterol [139]. Among these analogs, **CAP2** is as a bifunctional lipidoid that can be incorporated into LNPs devoid of cholesterol. These LNPs exhibit superior delivery performance for FLuc mRNA in vitro compared to MC3 LNPs (Table 3). Furthermore, microinjection of Dmc1 saRNA-loaded CAP2 LNPs into the seminiferous tubules restored spermatogenesis in Dmc1-deficient mice.

In summary, acyclic amino heads offer a wide variety of options for constructing ionizable lipidoids. Tail design appears to be especially important for modulating biodegradability, stability during storage, LNP delivery efficacy, and safety. Ligand-tethered structures can be used to target delivery or induce specific pharmacological effects. However, this tethering often yields LNPs with larger particle sizes (> 100 nm), which may limit applicability in humans as represented by the **SAL12-** and **DAL4**-based LNPs (Table 3) [140]. Moreover, the failure to translate **1A** LNP (Table 3) from in vitro to in vivo raises doubts about the potential for targeting lipidoids to be successfully utilized in medicines. Instead, recent studies have focused on selective organ-targeting (SORT) lipid integration for directed delivery by LNPs [141, 142]. The growing understanding of how structural characteristics affect LNP properties will be useful in expanding the usage of LNPs for delivery of RNA medicines, including vaccines, cancer therapeutics, gene therapies and others.

#### Computer-aided design and AI prediction of ionizable lipids

Despite significant research efforts, current designs of ionizable lipids only scratch the surface of the vast array of possible chemical structures. Nevertheless, constructing and screening a broader lipid library remains technically challenging, tedious and costly, even with high-throughput synthesis technologies and assays. Rather than massive screening studies, artificial intelligence (AI) and computational methods may soon revolutionize and expedite LNP component design, particularly for ionizable lipids. Along these lines, Jeong et al. has utilized machine learning (ML) algorithms and definitive screening design to optimize mRNA-LNP vaccines. The authors found that their artificial-neural-network design-of-experiment (ANN-DOE) model outperformed other ML models for this purpose [143]. In another study, Metwally et al. used ML techniques to predict the in vivo efficacy of siRNA LNPs and found that an ANN method made the most accurate predictions [144]. This application of AI allows researchers to rapidly evaluate a wide variety of potential ionizable lipids before dedicating resources to synthesis and biological testing. Thus, the approach can be used to improve allocation of materials and time when exploring LNP components. Another ML algorithm, LightGBM, was built and developed as prediction model for LNP screening based on a dataset of 325 mRNA-LNP vaccine formulations and their respective IgG titers. Notably, the ML model successfully identified critical substructures of ionizable lipids and precisely predicted that the in vivo efficacy of LNPs with DLin-MC3-DMA would be higher than those with SM-102, in agreement with experimental results [145].

Given the scarcity of published research on ML in lipid design, we will also discuss several studies that are disseminated on preprint servers. These papers provide information about potential AI applications that may be seen in future work. While the preprint manuscripts are discussed here, we must emphasize that the work has not been subjected to peer review at the time of writing this review. In one study, the Ghosh group utilized the LightGBM algorithm to examine LNP efficacy in a database of experimental studies [146]. From their analysis, the authors identified the number of outside carbons in ionizable lipids as a key feature influencing transfection efficiency. Guided by this insight, they next formulated LNPs with structurally modified SM-102 and ALC-0315 analogs. Experiments with the analogs validated their predictions, highlighting the potential of ML analyses to guide lipid design. In another preprint study, Xu et al. utilized the AI-Guided Ionizable Lipid Engineering (AGILE) platform for screening optimal LNP formulations [147]. AGILE is comprised of a pretrained neural network, which was fine-tuned with experimental data from screening of 1,200 ionizable lipids; the platform was used to predict the efficacies of a larger library of 12,000 candidates [148, 149]. Lipid hits from AGILE were synthesized and evaluated in biological systems, demonstrating its potential to expedite development of tailored LNPs [147]. While the combination of high-throughput screening and AI predictions shows great promise as a means of accelerating mRNA LNP design, the field is still nascent. For successful implementation of this approach, comprehensive open-access databases on LNP formulations and efficacies would be greatly beneficial. Such databases could be used to enhance computational prediction accuracy for diverse biomedical applications.

### Helper phospholipids

Endosomal escape is a major determinant of delivery efficiency for nucleic acid therapeutics. As such, helper phospholipids that enhance endosomal escape may be integrated into current LNP systems. Although helper phospholipids like DSPC and DOPE are commonly included in LNP formulations, these compounds have limitations of irreversible zwitterionic structures and hinderance of structural manipulation. In Liu et al.'s study, the authors introduced an ionizable phospholipid (iPhos) with a reversible zwitterion, which could potentially enable pH-triggered membrane rupture. An iPhos with a small zwitterionic head and multiple hydrophobic tails tended to take on a conical shape, allowing it to induce membrane phase transformation. Among 572 designed lipids, **9A1P9** (contains one tertiary amine, one phosphate group, and three alkyl tails) exhibited the highest efficacy in terms of extrahepatic delivery and selective organ targeting to the spleen, liver or lungs. LNPs formulated with **9A1P9** also demonstrated superior function according to mRNA expression and efficiency of CRISPR-Cas9 gene editing in liver and lung tissues [150]. In a recent study by the same research group, phospholipids with phosphoethanolamine (PE) head groups, like **POPE** and **4ME**, were found to enhance endosomal escape due to their fusogenic properties [151]. The same study also revealed that zwitterionic phospholipids primarily facilitated hepatic delivery, while negatively charged phospholipids (**BMP**) selectively targeted the spleen. Together, these findings contradict the traditional paradigm that phospholipids are simply ‘helper’ lipids and give reason for dedicated efforts toward optimization and development of novel phospholipids for mRNA delivery [152].

In recent years, only limited efforts have been made to rationally design helper phospholipids, with the focus shifting to phospholipid modifications. For instance, Butowska et al. developed a doxorubicin and siRNA co-delivery system by conjugating doxorubicin to siRNA-loaded LNPs [153]. The authors utilized a thiolated phospholipid to conjugate with doxorubicin, forming the modified phospholipid **DOX-EMCH-PTE** (Table 4). The optimized LNP carried siBcl-2 and was able to induce potent knockdown of Bcl-2 in Burkitts’ lymphoma (Raji) cells. Meanwhile, doxorubicin was delivered to the cell nucleus. These actions effectively inhibited tumor growth in vivo. Overall, this study demonstrates that combination phospholipid-linked chemotherapy and RNAi therapy may hold promise as a therapeutic approach.

**Table 4:**
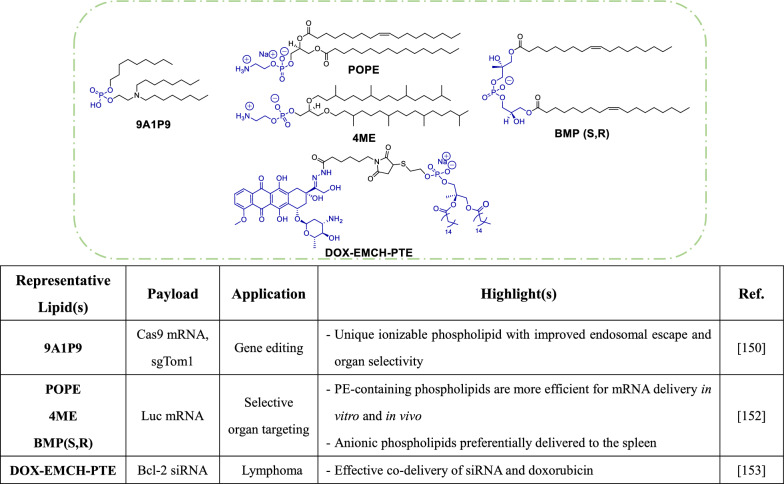
Helper phospholipids

### PEGylated lipids

Polyethylene glycol (PEG)-conjugated lipids play a crucial role in LNP formulations, preventing aggregation and keeping particle sizes between the approximate range of 50 to 150 nm. However, the inclusion of PEG has raised concerns regarding the compromises in activity. The tradeoffs associated with inclusion of PEG in LNPs are sometimes referred to as the 'PEG dilemma' [154]. PEG conjugation may hinder cellular uptake, potentially reducing the transfection efficiency of LNPs. Moreover, repeated systemic administration of PEG-based compounds may trigger the formation of anti-PEG antibodies, potentially resulting in unwanted hypersensitivity reactions. Considering these challenges, the Haas group has evaluated the substitution of PEG with polysarcosine (**pSar**) in mRNA-LNPs (Table 5). Similar to the PEG system, particle sizes tended to decrease with increasing **pSar** concentration in LNPs [154]. Additionally, LNPs containing **pSar** were typically larger than those containing PEG lipids at a similar molar fraction. Notably, **pSar**-LNPs had poorer performance than PEG-LNPs, according to luciferase expression, but the performance increased with higher **pSar** lipid fraction. This observation may be attributed to the chemical structure of **pSar**, as the secondary amine may facilitate electrostatic binding with negatively charged cellular membranes [154]. In vivo studies on pSar-LNPs revealed that liver and spleen were the tissues with the highest mRNA expression. However, LNPs that included **pSar** with shorter lengths tended to target the spleen specifically. The authors also addressed questions regarding the in vivo safety profile of the modified LNPs. Notably, administration of **pSar-**LNPs to mice induced similar or lower levels of toxicity indicators (i.e., AST, ALT, LDH and total bilirubin) compared to PEG-LNPs; no significant changes were observed in the body weights of treated mice. Furthermore, LNPs formulated with **pSar23** showed less cytokine induction compared to PEG-LNPs, suggesting that the substitution of PEG with **pSar** in LNPs is not likely to increase toxicity [154]. Offering another solution to the ‘PEG dilemma’, Yu and colleagues explored poly(ethyl ethylene phosphate) (**PEEP**) as a potential alternative to PEG, as **PEEP** exhibits superior water solubility, biocompatibility and stealth effects [155]. Ovalbumin mRNA-LNPs were constructed with **DSG-PEEP** (Table 5), and these LNPs exhibited similar morphology and function to LNPs constructed with DSPE-PEG. In vivo, **PEEP**ylated LNPs elicited potent antigen-specific T cell responses and effectively suppressed tumor growth, demonstrating comparable efficacy to PEG-based LNPs [155]. Berger and others studied the potential of amphiphilic poly(*N*-methyl-*N*-vinylacetamide) (**PNMVA**) as a PEG-lipid alternative for siRNA delivery [156]. Their results revealed that **DSPE-PNMVA** efficiently integrates into lipoplexes and LNP membranes, and it has stealth properties similar to DSPE-PEG. Lipoplexes comprised of **DSPE-PNMVA**_**24**_ did not show any significant toxicity in vitro or in vivo, unlike DSPE-PEG and **DSPE-PNMVA**_**50**_. Further evaluation also suggested that **DSPE-PNMVA**_**24**_ lipoplexes are less immunogenic than DSPE-PEG-containing LNPs, since serum cytokine levels in treated mice were comparable to those in the PBS control group [156]. Incorporation of DSPE-PEG into LNPs loaded with siGFP showed efficient inhibition of GFP fluorescence (> 65% inhibition), however in the presence of serum, the LNPs conferred markedly less (< 45%) inhibition. In contrast, LNPs with **DSPE-PNMVA**_**24**_ caused higher inhibition (~ 80%), even in the presence of serum. The results with **DSPE-PNMVA**_**24**_ LNPs were comparable to those of the Lipofectamine positive control [156].

**Table 5:**
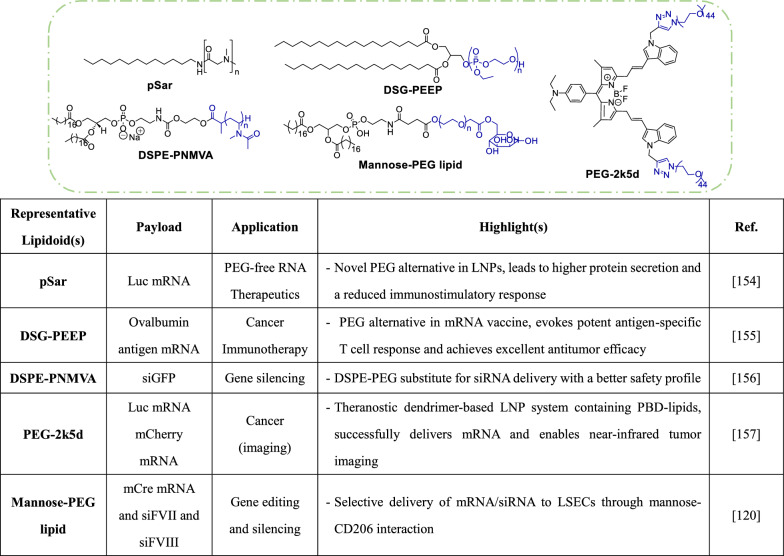
PEGylated Lipids

In contrast to strategies utilizing entirely new polymers to replace PEG, other studies have explored modified PEGylated lipids. The Siegwart group investigated PEGylated BODIPY dyes (**PBDs**), which structurally resemble the conventional PEG-lipids used in LNPs [157]. The authors proposed that **PBDs** could serve as desirable surface-stabilizing agents in LNPs, given that their pH-responsiveness may aid in the release of mRNA during endosome maturation. LNPs formulated with pH-responsive **PBD-lipids** could mediate efficient delivery of mRNA to cells, resulting in significantly higher cytoplasmic protein production (~ 5- to 35-fold increases) compared to PEG-DMG LNPs. These **PBD**-based LNPs also exhibit better mRNA delivery in vivo, with the highest protein expression observed in the liver of animals treated with LNPs that had a pKa ~ 6.3. In a different study, **PEG2k5d** (Table 5) LNPs could be used in a theranostic application to achieve robust mRNA expression in tumors while also enabling pH-responsive near-infrared tumor imaging [157]. Altering the functionality of PEG lipids in LNPs can influence tissue targeting specificity. Lee et al. achieved selective targeting of LSECs by incorporating mannose into the PEG lipid [120]. This design capitalizes on the high expression of the mannose receptor on human and murine LSECs. Compared to unmodified PEG-containing LNPs, the **mannose-PEG**-containing LNPs demonstrated enhanced transfection efficiency in LSECs. Moreover, inclusion of the **mannose-PEG**-lipid in siRNA-loaded LNPs led to more pronounced FVIII inhibition in LSECs, while galactose-PEG-lipid LNPs showed no significant effect [120].

### Steroids

Enhancement of mRNA delivery through the development of novel ionizable lipids has garnered considerable attention. However, cholesterol, a critical component of lipid nanoparticles (LNPs) and a highly prevalent steroid primarily produced in the liver, has received comparatively less rigorous investigation in recent studies. The inclusion of cholesterol in LNP formulations improves efficacy by enhancing membrane fusion. To better understand how cholesterol can impact transfection efficiency, the Sahay group conducted a comprehensive SAR analysis on cholesterol analogues in mRNA-LNPs [158]. Their study revealed that inclusion of different C-24 alkyl phytosterols in LNPs can enhance gene transfection, with critical structural factors including the alkyl tail length, sterol ring flexibility, and the polar OH group. In particular, **β-sitosterol**, a plant sterol, was identified as an effective constituent for boosting transfection with LNPs containing different ionizable lipid types and nucleic acid payloads in various cell types (Table 6). Notably, **β-sitosterol**-LNPs exhibited higher cellular uptake and retention of payloads than cholesterol-LNPs. This difference was attributed to potential efflux via cholesterol transporters on late endosomes. Thus, it appears that replacing cholesterol with **β-sitosterol** may mitigate efflux, enhance cellular retention, and ultimately increase gene expression. Moreover, the increased fragility of **β-sitosterol**-LNPs within cells may promote fusion with the endosomal membrane, further contributing to their superior performance [158]. These findings underscore the pivotal role of cholesterol in subcellular mRNA-LNP transport and suggest further research into the design and development of this crucial LNP component would be beneficial. One recent study on the mechanisms underlying enhanced mRNA delivery by LNPs with alternative phytosterols suggested that the effect may involve endosomal recycling mechanisms mediated by the Niemann-Pick C1 (NPC1) enzyme [159]. Structural modifications to cholesterol may reduce recognition by NPC1, leading to improved cellular retention and increased mRNA expression. To further explore this idea, a library of LNPs incorporating various **hydroxycholesterols** (Table 6) was assessed in terms of mRNA delivery to T cells [159]. It was hypothesized that addition of a polar hydroxyl group to cholesterol may alter its binding to NPC1, potentially reducing NPC1 recognition during endosomal trafficking of LNPs. Experimental results showed that substituting **hydroxycholesterol** into LNP formulations affected particle stability, with body-modified cholesterols yielding stable LNPs and tail-hydroxylated cholesterols causing minimal stability changes. Since cholesterol recognition by membrane proteins is crucial for endocytosis, the study also characterized the effects of partial substitutions on mRNA delivery efficiency. LNPs with moderate proportions of substitutions (25% and 50%) exhibited enhanced transfection efficiency compared to those with lower (12.5%) or higher (100%) substitution rates. Among the tested formulations, **A1-25** (25% substitution with **7α-hydroxycholesterol**) and **A1-50** exhibited improved transfection efficiencies without significant changes in LNP toxicity in vitro. Further analysis of endosomal trafficking revealed that hydroxylation increased late endosome production and reduced endosomal recycling [159].

**Table 6.**
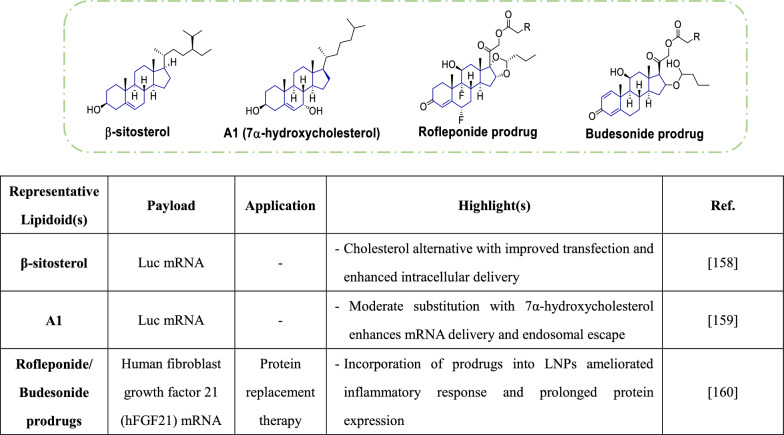
Steroids

Considerable advances have been made in developing potent and well-tolerated LNPs for mRNA delivery via intravenous or intramuscular injection. However, subcutaneous administration remains challenging, as local retention of LNPs and mRNA payloads can lead to adverse inflammatory responses [160]. To address this issue, Davies and colleagues explored the incorporation of hydrophobic prodrugs of anti-inflammatory steroids (i.e., **rofleponide** and **budesonide**) (Table 6) into mRNA-loaded LNPs. With this strategy, the authors aimed to mitigate inflammation during systemic protein replacement therapy with human fibroblast growth factor 21 (hFGF21) [160]. Various aliphatic ester prodrugs with differing chain lengths (C5: **rofleponide** only, C8: **budesonide** only, C14, C16, and C18) were synthesized to increase lipophilicity and enhance encapsulation in LNPs. The experimental results demonstrated that integrating **rofleponide** and **budesonide** prodrugs into LNPs reduced both local (edema) and systemic inflammatory responses, with greater reductions observed for longer carbon chain ester prodrugs (C16 and C18). Interestingly, the inclusion of anti-inflammatory steroid prodrugs in LNPs also prolonged protein expression and increased plasma protein exposures. Pharmacokinetic studies indicated that increasing alkyl chain length led to a prolonged half-life, suggesting that an inverse relationship between ester hydrolysis rate and alkyl chain length may greatly influence retention at the administration site [160]. These findings underscore the role of steroids in establishing the versatility and adaptability of LNPs for delivery of nucleic acid therapeutics.

Owing to the demonstrated success of mRNA-LNP COVID-19 vaccines (Comirnaty™ and Spikevax™) during the pandemic, a global spotlight has been shined on the potential of the LNP platform in development of future therapeutics [161, 162]. Current trends in clinical trials suggest that nucleic acid therapeutics will continue to dominate among other treatment modalities in the next decade, cementing the position of LNPs as one of the most relevant drug delivery systems to date [163]. Although the efficiency and applicability of LNP technology are widely heralded, it is also crucial to understand and regulate the potential adverse effects associated with this technology. For instance, several reported cases of anaphylaxis, pseudo-allergy, and hypersensitivity reactions have been attributed to the inclusion of PEG in mRNA-LNP vaccine formulations [164–168]. Post-vaccination myocarditis and pericarditis have also been documented and are hypothesized to be induced by elevated levels of circulating unbound vaccine-derived spike protein [169–171]. In rare cases, the mRNA-LNP vaccines may also be recognized as self-antigens, which may trigger autoimmune diseases [172]. Therefore, a balance between immunogenicity and reactogenicity must be established in the development of mRNA-LNP vaccines. To modulate the immunogenicity of mRNA-LNPs, various strategies may be utilized, including (1) optimization of LNP components or formulations, (2) inclusion of adjuvants, and (3) modulation of the administration mode [164]. Despite the fact that there are currently unknown innate immune mechanisms and interplay between immune components that contribute to the adverse effects of LNPs, the substantial benefits of using LNP technology for mRNA therapeutics outweigh the associated clinical risks. Continued research efforts are expected to provide more complete knowledge about the potential immunological effects of LNPs and possibly result in a major breakthrough in personalized mRNA-based vaccines for various diseases.

## Targeting strategies for mRNA delivery

The architecture of solid tumors poses a great challenge for effective delivery of small molecule drugs and mRNA-based therapeutics, potentially limiting the therapeutic efficacy of many treatments. Within solid tumors, the microenvironment is often characterized by high cell density, irregular blood vessel structures, elevated interstitial pressure, and an acidic milieu, which collectively impede drug penetration. These physiological barriers may prevent therapeutics from accessing solid tumors and lead to suboptimal treatment outcomes. Matsumura and Maeda's pioneering work demonstrated that nanoparticles can extravasate through inherently leaky and loosely compacted tumor vasculature, remaining within the tumor space due to poor lymphatic drainage; this effect is known as Enhanced Permeability and Retention (EPR) [173]. Despite the EPR-mediated enhancement of passive targeting, a modest < twofold increase in nanodrug entry was measured in tumors compared to normal organs [174]. Thus, the intratumor drug concentrations achievable with passively targeted nanoparticles may be inadequate for effective treatment of most cancers [175, 176].

### New modality of mRNA-based vaccines and drugs: lipid compositions and ligand targeting

The rapid development and high efficacy of mRNA-based COVID-19 vaccines stimulated great interest in mRNA-based drugs or vaccines against various infectious and immunological diseases. In addition, the ability to chemically synthesize stable mRNA was a breakthrough that expanded the drug development potential for mRNA technology. Since IVT-generated mRNAs can be directly translated into therapeutic proteins, mRNA is widely considered a highly promising therapeutic modality in the pharmaceutical industry [177]. The potential of mRNA-based medicines is not only limited to vaccines for infectious diseases, and the technology may prove to be an excellent medium for gene and protein therapies as well. An advantage of mRNA-based drugs is that they are not subject to the high production costs associated with antibody-based drugs. Another advantage is that optimized delivery strategies may improve therapeutic efficacy by allowing for specific delivery of therapeutic nucleic acids to target cells [178]. For example, an mRNA encoding Palivizumab was translated into neutralizing antibodies in vivo and showed better therapeutic efficacy than Palivizumab in terms of RSV 7 inhibition [179]. In addition, mRNA-based cancer vaccines can be made to target TSAs, TAAs, and immunomodulatory factors that are known to play essential roles in disease [180]. Many well-characterized tumor-modulatory factors play roles in cancer immunity, and one FDA-approved technology that exploits this fact is adoptive cell transfer, an advanced and personalized technology used to treat leukemia [181]. Modifications of this technology are being explored to treat solid tumors, as Beatty et al. developed an mRNA encoding chimeric antigen receptor (CAR) for mesothelin to treat pancreatic ductal adenocarcinoma (PDAC) in a Phase 1 study [182]; study completion was reported in October 2015 (ClinicalTrials.gov: NCT01355965). Additionally, an anti-BCMA mRNA-transfected CAR-T therapy demonstrated primary efficacy in patients with multiple myeloma [183] in a phase 1/2 study completed in December 2021 (ClinicalTrials.gov: NCT03448978). In another study, Sahin’s research group has pioneered a novel approach involving the administration of anti-claudin 6 (CLDN6) CAR-T therapy in conjunction with LNP-encapsulated CLDN6 mRNA vaccine (CARVac). This therapy was administered to 22 patients with CLDN6-positive solid tumors during a phase 1 trail [184]. The therapeutic regimen demonstrated notable anti-tumor efficacy at the study's endpoint, laying the foundation for a promising new treatment utilizing CAR-T therapy for solid tumors.

Despite ample evidence that mRNAs may be useful to treat as yet incurable diseases, the clinical translation of mRNA-based drugs for protein replacement and gene therapies has been limited. In contrast to other therapeutic modalities, such as delivery of nucleic acid cargos to targeted cell populations in order to limit off-target effects and cytotoxicity [25, 185]. One challenge in this regard is that targeted delivery of mRNA requires protecting mRNA from degradation, which is usually achieved by wrapping it in a nanocarrier. For this purpose, the most advanced nanomaterial to date is ionizable LNPs [11]. Targeted delivery of mRNA-LNPs to a specific site can be achieved by active or passive targeting [2, 186, 187]. The approach of passive targeting is largely dependent on EPR [173, 188]. However, in vivo delivery of nucleic acid cargos via passive targeting is difficult to modulate. Therefore, active targeting may be a better option to achieve well-controlled delivery. In general, active targeting involves the use of targeting ligands, such as full antibodies, antibody fragments or ligand peptides [189–191]. These targeting ligands allow for delivery of mRNA-LNPs into specific cell types by binding to receptors on the cell surface [187].

In addition to ligand-mediated targeting, different modified ionizable lipids can facilitate delivery of mRNAs to specific cell types or organs. One recent publication compared a series of synthesized ionizable LNPs in terms of their ability to target liver sinusoidal endothelial cells (LSECs); the LNPs had varied PEG percentages and nanoparticle sizes [120]. In another study, Siegwart's group designed Selective ORgan Targeting (SORT) nanoparticles by incorporating certain molecules into lipid nanoparticles comprised of ionizable lipids, cholesterol, DSPC and PEG. The resulting SORT nanoparticles tuned mRNA release based on modulation of internal charge and thereby facilitated delivery to specific tissue types [141]. Since tuning ionizable lipids based on organ or cell type may be difficult to achieve in clinical practice, targeting of LNPs with specific ligands may be the best strategy. Ligand-mediated targeting has been shown to mediate delivery nucleic acid cargos to specific cell types overexpressing the receptor [192]. Targeting of mRNA-LNPs can be achieved by introducing the targeting ligands during LNP production or by post-insertion of targeting ligands into the mRNA-LNP. Recent studies have shown that different targeting ligands can be inserted into mRNA-LNPs to specifically target immune cells, cardiac cells and liver cells. The research field of targeting mRNA-LNPs is still in its infancy, with relatively few publications available. Table 7 summarizes the different ligands utilized to target mRNA-LNPs to different cell types.

**Table 7:** Targeting mRNA-LNPs to different cell types

LNP composition	Targeting ligand	Conjugation strategy	mRNA	Ref.
MC3, DSPC, Cholesterol, DMG-PEG, and DSPE-PEG (50:10.5:38:1.4:0.1 molar ratio)	Anti-Ly6c mAbs	Modular targeting platform named ASSET (Anchored Secondary scFv Enabling Targeting) was used	Interleukin-10 (IL-10) mRNA; treatment of inflammatory bowel disease	[193]
MC3, DSPC, Cholesterol, DMG-PEG, and DSPE-PEG Mal (50:10:38:1.5:0.5 molar ratio)	Anti-CD4 antibody	DTT-reduced IgG was post-inserted into maleimide-functionalized LNPs	Cre recombinase-encoding mRNA	[194]
Ionizable cationic lipid (proprietary to Acuitas), phosphatidylcholine, cholesterol, PEG-lipid (50:10:38.5:1.5 molar ratio)	Anti-PECAM-1 antibody	Targeting LNP was generated via SATA–maleimide conjugation chemistry	Luciferase mRNA	[195]
Ionizable lipid, DSPC, cholesterol, DMG-PEG, and DSPE-PEG (50:10.5:38:1.4:0.1 molar ratio)	EGFR-antibody	ASSET (Anchored Secondary scFv Enabling Targeting) linker system	Cas9 mRNA and sgRNAs; CRISPR-LNPs against PLK1	[196]
MC3, DSPC, DSPE-PEG2k, DSPE-PEG5k-Mal, Cholesterol (50:10:1.5:0.5:38, molar ratio)	Anti-CD3 antibody	TCEP-reduced IgG was post-inserted into maleimide-functionalized LNPs	mCherry mRNA	[197]
Ionizable cationic lipid, phosphatidylcholine, cholesterol and polyethylene glycol-lipid	Anti-CD5 antibody	Targeting LNP was generated via SATA–maleimide conjugation chemistry	mRNA encoding a CAR designed against fibroblast activation protein (FAP)	[198]

### Targeted delivery systems for mRNA vaccines and drugs

The therapeutic applications of mRNA are rapidly advancing in a variety of areas, and it is becoming more important to be able to target the nucleic acid to different cell types and organs. Currently, LNPs are used to encapsulate mRNA payloads into a particle core that stabilizes, and controls release and distribution, which improves uptake by immune cells and prevents degradation [199]. However, it remains challenging to design LNPs that can specifically target appropriate cells and pass through different physiological and biological barriers.

#### Passive targeting

Passive targeting is largely affected by the physical properties of an LNP, such as its size, shape and surface charge distribution, which may allow it to deposit cargo within the tumor milieu [187]. This ability of LNPs to accumulate in cancer tissues is mainly derived from the EPR effect [200]. For instance, LNPs within the size range of 20–200 nm are generally permeable and retained within tumors via the EPR effect. Since tumor microenvironments are often hypoxic, rapid but defective angiogenesis tends to occur within the tissue. This allows LNPs to readily pass through the blood vessels due to enhanced vascular permeability and accumulate in the tissue [173, 201]. At the same time, inefficient lymphatic drainage from the tumor results in the enhanced retention of LNPs [174]. This approach has been successfully used in clinical applications. In 1995, Doxil® became the first LNP drug to be authorized by the US FDA. Doxil is passively targeted via EPR to tumors, where its payload of doxorubicin is released. The drug is approved for ovarian cancer and AIDS-related Kaposi’s Sarcoma treatment [175]. To extend this approach to mRNA-based therapeutics, Kranz et al. generated RNA-lipoplexes with a negative charge that passively target to dendritic cells after intravenous administration. These lipoplexes were shown to induce type-1 interferon-mediated immunity and restrict aggressive tumors in a mouse model [176].

#### Endogenous targeting

One approach involves modified LNPs to interact with endogenous proteins that are present in specific tissues or cell types. These interactions can enhance the uptake of LNPs by target cells, thereby improving the delivery of therapeutic payloads such as nucleic acids or drugs. Since surface charge, size and lipid components of nanoparticles have been reported to modulate delivery to specific organs, researchers have created some modified LNPs that interact with endogenous proteins to provide tissue specificity [120, 141]. When LNPs have a neutral surface charge, the formation of complexes between LNPs and apoE facilitates transport to the liver and uptake by hepatocytes and hepatoma cells through LDL receptors [202]. LNPs conjugated with apoE are successfully targeted the central nervous system, crossing the blood–brain barrier and releasing an anticholinergic drug for treatment of Alzheimer’s disease [203]. In contrast, LNPs with anionic charge can be utilized to facilitate delivery to spleen via adsorption to β2-glycoprotein I [204, 205]. In addition, the different phospholipids not only affect LNP tissue-tropism but may also interfere with the biological process of protein translation [206].

#### Active targeting

Active targeting involves decoration of the LNP surface with ligands that specifically interact with highly expressed receptors on target cells. Several types of ligands, such as antibodies, peptides, aptamers, polysaccharides (or glycans) and small molecules, may be used to improve binding affinities and facilitate the cellular uptake of LNPs for cancer therapy [199]. An efficient ligand-receptor interaction depends mainly on the ligand binding affinity and density on the surface of LNPs. Full-length antibodies containing Fc domains are frequently used for targeting, as these ligands show specific binding and therapeutic capabilities. The Fc domain of an antibody enables triggering of Fc-mediated effector functions, and it binds with the neonatal Fc receptor FcRn to prolong its half-life in circulation [207, 208]. However, antibodies are macromolecules, and their large size limits the density that may be conjugated on the surface of LNPs; the large size may also interfere with diffusion into the tumor interstitium, thereby reducing the therapeutic efficacy [209]. To overcome these limitations of large targeting molecules, many studies have been conducted on antibody fragments lacking the Fc region, such as antigen-binding fragments (Fab) and single-chain variable fragments (scFv). These fragments have smaller molecular weights but retain high affinity to target cells and exhibit high penetration efficiency [210]. Although scFv and Fab fragments have both been applied in LNPs constructs [211, 212], scFvs are thermally instable, which introduces difficulties in manufacturing [213]. Fabs are more thermally stable, so these fragments can provide both the stability and affinity essential for high neutralizing capacity [211]. The addition of PEG moieties (PEGylation) and serum albumin can be used to enhance the half-life of an LNP with conjugated antibody fragments [210]. Aside from antibodies, targeting peptides can be identified from in vivo biopanning of a M13 bacteriophage-display peptide library [191]. The utility of peptide-conjugated LNPs was demonstrated in a study showing successful delivery of mRNA to the retina in a potential treatment for inherited blindness [214]. In addition, the density of ligands on the surface of the LNPs can be enhanced by incorporation of the RGD tripeptide, which can lead to multivalent enhancement of affinity and extend the LNP half-life [215]. LNPs with incorporated trivalent N-acetylgalactosamine (GalNAc) are effectively recognized and processed by asialoglycoprotein receptor (ASGPR) on hepatocytes in an apoE-independent mechanism [202].

### Strategies to promote endosome escape of LNPs

**Fig. 4:**
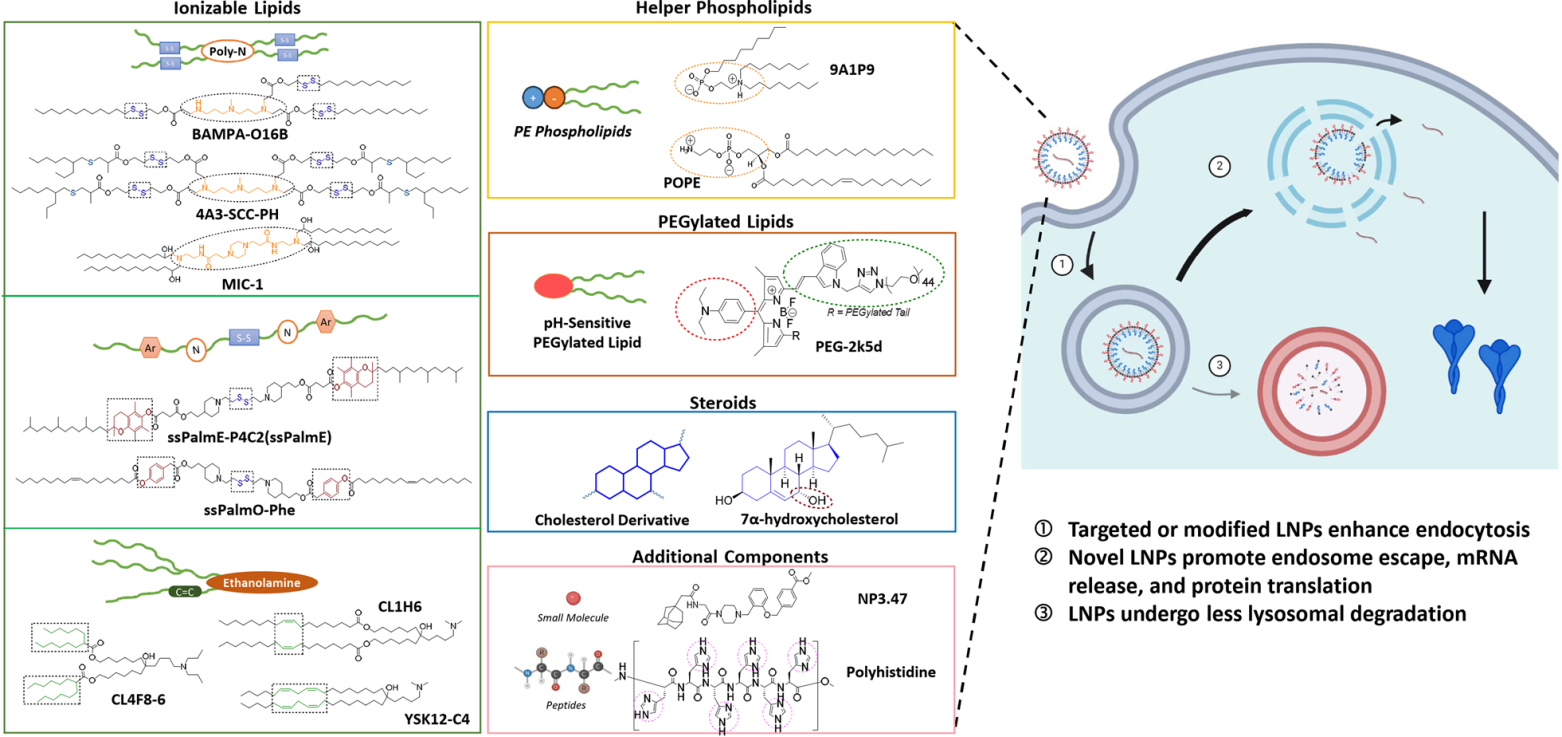
Strategies to facilitate endosomal escape of LNPs for enhanced mRNA release and protein translation. LNPs are taken up into cells via endocytosis. Targeted or modified LNPs can enhance endocytosis. Innovative ionizable lipid structures used in the formulation of LNPs can facilitate endosomal escape and release of the payload into the cytosol. The integration of cutting-edge ionizable lipid structures within LNPs can significantly enhance endosomal escape, facilitating the efficient release of the encapsulated payload into the cytosol. Novel ionizable lipids commonly feature distinctive characteristics, such as a polyamine head group with a pKa around 6.5, incorporation of aromatic ring moieties in linker chains, and diverse tail compositions (e.g., bioreducible disulfide, branched, or unsaturated tails). Different helper lipids, such as PE-containing phospholipids, pH-sensitive PEGylated lipids, and hydroxylated cholesterol derivatives can contribute to endosomal escape as well. Integration of other materials, such as the small molecule inhibitor NP3.47 or polyhistidine peptides can also promote this process. Novel LNPs with these features have been shown to enhance endosomal escape and reduce lysosomal degradation of mRNA cargoes, which eventually promotes mRNA escape from endosomes and facilitates mRNA release, ultimately increasing translation of the encoded protein. Created with BioRender.com

LNPs are efficiently endocytosed into cells and transported to endosomal compartments. To further deliver the mRNA cargo into the cytosolic region, LNPs must escape from endosomes. Within the acidic endosomal environment, the ionizable lipids in LNPs are protonated and become positively charged, which allows the lipids to bind negatively charged lipid molecules on endosomal membrane. This interaction triggers phase transition and fusion of the LNP with the endosomal membrane, consequently releasing the nucleic acid cargo into the cytosol. A major limitation of this approach is that most of the endocytosed LNPs are eventually guided to lysosomes for degradation, and only a very small portion (about 2%) successfully escape from endosomes to deliver the nucleic acid cargo. Several strategies have potential to overcome this limitation, including the development of novel ionizable lipids, incorporation of helper lipids and inclusion of other new materials (Fig. 4). With these approaches, researchers expect to enhance endosomal escape of LNPs and increase the efficiency of nucleic acid delivery by LNPs.

In one study, Liu et al. designed and screened a series of cationic LNPs with different ionizable amine headgroups; they identified a cationic lipid called BAMPA-O16B, which has a pKa of about 6.5 and is superior in triggering endosomal escape and siRNA delivery [216]. The results of their study indicate that the amine headgroup has a major influence on endosomal escape and siRNA delivery efficiency. Furthermore, Tanaka et al. observed that the insertion of an aromatic ring into the hydrophobic scaffold of ionizable lipids can increase endosomal escape and enhance mRNA expression in vitro and in vivo. Chen et al. also reported that disulfide bond-bridged linkers of ionizable lipids contribute to superior endosomal escape and rapid mRNA release [103]. Meanwhile, Nakamura et al. offered evidence that two cationic lipids (YSK12-C4 and CL1H6), each of which contain two double bonds within their hydrophobic tails, can promote endosomal escape of LNPs [217]. Lee et al. further found that an unsaturated tail of the ionizable lipid promotes endosomal escape and contributes to enhanced in vivo mRNA delivery [105]. Moreover, Hashiba et al. offered evidence that branched tails of ionizable lipids promote LNP endosomal escape and delivery of mRNA [127].

In addition to developing novel ionizable lipids, Munson et al. observed that replacing the cholesterol, the helper lipid of LNPs, with beta-sitosterol (contains a longer alkyl side chain) promotes endosomal escape and mRNA delivery. Furthermore, another study showed that replacing the neutral helper lipid DMG-PEG with a negatively charged DMPE-PEG helper lipid can also enhance endosomal escape and mRNA delivery [218]. Álvarez-Benedicto et al. also provided evidence that substituting the DSPC phospholipid with phosphoethanolamine-containing DOPE enhances LNP endosomal escape and mRNA delivery efficiency [152].

In addition to modulating lipid structure and composition, researchers have incorporated new materials into LNPs in an effort to enhance endosomal escape and delivery efficiency. For instance, Wang et al. integrated a small compound known as NP 3.47 (an inhibitor of the Niemann-Pick type C-1 protein, which regulates intracellular cholesterol trafficking) into LNPs. Administration of these NP3.47-incorporated LNPs resulted in the accumulation of the LNPs within late endosomes or lysosomes, thereby augmenting the silencing potency of the siRNA cargo. [219]. Moreover, Kim et al. reported that the level of endosomal escape and knockdown efficiency was increased when cells were treated with LNPs containing polyhistidine polypeptides. As the pKa of polyhistidine polypeptides is 6.0–6.4 and the endosomal pH is about 6.0, the authors hypothesized that polyhistidine polypeptides are protonated in acidic endosomal compartments and subsequently destabilize endosomal membranes, which promotes polyhistidine-loaded LNP escape from the endosomal compartment [220].

Poor endosome escape of LNPs makes it necessary to apply higher doses in order to achieve therapeutic activity. However, treatment with high doses of LNPs may cause tissue damage via direct cytotoxicity and inflammation [221]. Here, we summarize strategies to generate novel LNPs with improved endosome escape capabilities, such as inclusion of novel ionizable lipids, different helper lipids or new materials. By enhancing endosomal escape of LNPs, mRNA delivery to the cytosol and protein expression are both improved. These strategies to enhance endosomal escape are expected to facilitate design of LNPs with higher efficiency of payload delivery and lower toxicity in vivo, which can improve the clinical characteristics of mRNA-based medicines.

### Immune cell targeting

Immune cells are categorized in two groups, innate and adaptive. Innate immune cells include macrophages, dendritic cells (DCs) and natural killer (NK) cells, while adaptive immune cells include T cells and B cells. Every type of immune cell makes an important contribution to the prevention of disease and defense of the body to both external and endogenous threats. Thus, delivery of mRNA encoding certain genes to any type of immune cells has the potential to augment cellular function and improve immunological defenses. In some cases, targeted delivery of therapeutic genes can be achieved by simply altering the surface charge, composition and structure of lipids in mRNA-LNPs. Alternatively, conjugation of targeting ligands on the LNP surface may be able to minimize off-target effects and mediate direct targeting of specific cells. Growing proficiency in bimolecular technology has allowed researchers to design targeting moieties for delivery of mRNA to appropriate target cells. For instance, DCs can be targeted with DEC205 scFv-coated LNPs. DEC205 is highly expressed on CD8α + DCs, and direct targeting of DCs via DEC205 enhances cellular uptake of a target gene [189]. Additionally, Wilson et al. demonstrated that the conjugation of mannose moieties to the surface of a polymer enhances uptake of the polymer into mannose receptor-expressing DCs [222]. Apart from active targeting of DCs, other factors such as physiochemical properties of LNPs affect the uptake of mRNA into DCs such as particle size, surface charge, and composition of lipids. Kranz et al. tune the delivery of RNA-lipoplexes by adjusting the surface charge of nanoparticles. They found that negatively charge nanoparticles can uptake DCs more likely than positively charged nanoparticles [176]. Similarly, in another study authors found that the size of the nanoparticles influences mRNA-LNP uptake into DCs. The particle size ranges from 200 to 500 nm and is more likely to interact with splenic DCs than lower particle sizes [223].

T cells possess an important function to treat various diseases, manipulating T cells enhances the immune potential. Targeting T cells for delivery of mRNA cargo can help to activate cytokines, manipulate the tumor microenvironment, activate immune cells, and modify T cells such as generation of chimeric antigen receptors (CARs). Simply the process of CAR-T therapy in vivo delivery of the target gene into T cells has been highly studied in the past few years. Decorating the nanoparticle surface with targeting ligands against CD3 [224], CD4 [225], CD5 [198], CD7 [226] reduces the off-targeting effects and enhances the therapeutic effect. Rurik et al. show that in vivo targeting of T cells by CD5 antibody shows only 20% positive expression of CAR-T cells, however, this much expression is enough to treat heart fibrosis in a murine model [198]. Later, the same group of researchers performed an extensive study to enhance the targeting ability of T cells in the presence of cytokines. They chose three cytokines, including interleukin-2 (IL-2), interleukin-7 (IL-7), and interleukin-15 (IL-15), and treated mice prior to injection with CD5-targeting LNP-mRNA. The results revealed that treatment with IL7 only enhanced the cellular uptake of CD5-LNP-mRNA in the mice model. The upregulation of mRNA expression may be because of the expansion of CD4 + and CD8 + T cells [227]. As a result of all these advances as well as the development of targeted delivery of mRNA into immune cells, diverse diseases may be treated successfully in the clinic shortly.

### Tumor targeting

Much research on mRNA-based cancer therapy has focused on the utilization of immunotherapeutic proteins like cytokines and costimulatory receptors. For instance, Hotz et al. formulated an innovative saline mixture containing four m1Ψ-modified mRNAs and administered the treatment by intratumoral injection [228]. The naked mRNAs encoded single-chain interleukin-12 (IL-12), interferon (IFN)-α, granulocyte–macrophage colony-stimulating factor (GM-CSF), and IL-15 sushi [228]. This combination therapy induced robust antitumor immune responses, which led to tumor regression in multiple mouse models. In two related studies, intratumoral administration of IL-12 mRNA either encapsulated in LNPs [134] or by inhalation of extracellular vesicles [229], induced a highly inflammatory tumor microenvironment and effectively stimulated both innate and adaptive systemic antitumor immunity. These data support the further development of cytokine-encoding mRNAs as treatments for cancer. In another key contribution to the field, Li et al. showed that a certain biomimetic nanoparticle could effectively deliver OX-40 costimulatory receptor mRNA to tumor-infiltrating T cells [133]. This approach was able to enhance the antitumor effects of its target antibody, anti-OX40. Most recently, intravenous administration of LNP-encapsulated mRNAs encoding SIRPα-Fc-CD40L and TIGIT-Fc-LIGHT led to in vivo production of hexameric proteins. These proteins augmented the population of antigen-specific CD8 + T cells within tumors, enhanced IL-2 expression, and markedly improved the efficacy of anti-programmed death-ligand 1 (PD-L1) antibodies in a combination therapy, as indicated by prolonged survival of CT26-bearing BALB/c mice [230]. At present, a broad spectrum of targeted mRNA-LNP technologies holds promise to transform the field of oncology, though many of these applications are still under evaluation in preclinical studies.

Immunotoxins comprise an intriguing biological drug class with applications in cancer treatment. An example immunotoxin is moxetumomab pasudotox (anti-CD22 Fv fused to PE38), which is approved by the US FDA for leukemia therapy [231]. Although immunotoxins have made their way into clinical use, the molecules have inherent characteristics that may contribute to resistance [232]. The mechanism of action for immunotoxins involves binding to target receptors, followed by internalization and intracellular trafficking of the toxins. These processes are all susceptible to resistance mechanisms, such as reduced cell-surface antigen presentation, impaired toxin processing, or toxin cleavage within the lysosome. To overcome at least some of these resistance mechanisms, a possible strategy could be LNP-encapsulation of mRNAs encoding the immunotoxin proteins [233]. Taken together, these studies on mRNA-LNP delivery of cytokines, costimulatory receptors and immunotoxins showcase promising strategies in which the immunotherapeutic proteins may be harnessed or targeted to provide effective treatments. Moreover, targeting studies on specific LNP compositions (without the use of ligands; see *“*Design and development of ionizable and cationic lipids” section) have greatly improved the efficiency of mRNA delivery to tumors and immune cells in mouse models upon systemic administration [234, 235].

Systemic administration of mRNA-LNPs encoding tumor suppressors has been shown to restore expression of the tumor-suppressive protein. For example, this approach has been taken to deliver the well-known tumor suppressor p53, which is mutated in ~ 50% of all human cancers [236]. Such administration of p53 mRNA-LNPs represents a promising approach for restoring p53 protein levels in cancer cells. This strategy has demonstrated efficacy in inhibiting the growth of p53-null hepatocellular carcinoma and NSCLC cells in vitro and in various animal models [237]. The mechanism of action involves induction of cell cycle arrest and apoptosis in tumor cells, which ultimately impedes tumor progression. Similarly, PTEN is a widely recognized tumor suppressor gene that is frequently lost or mutated in metastatic castration-resistant prostate cancers and numerous other human malignancies. Reintroduction of PTEN to PTEN-null prostate cancer cells using mRNA-LNPs results in significant inhibition of tumor cell growth both in vitro and in vivo [238]. Importantly, this effect can be observed after systemic delivery of the mRNA-LNPs in multiple mouse models of prostate cancer.

**Fig. 5:**
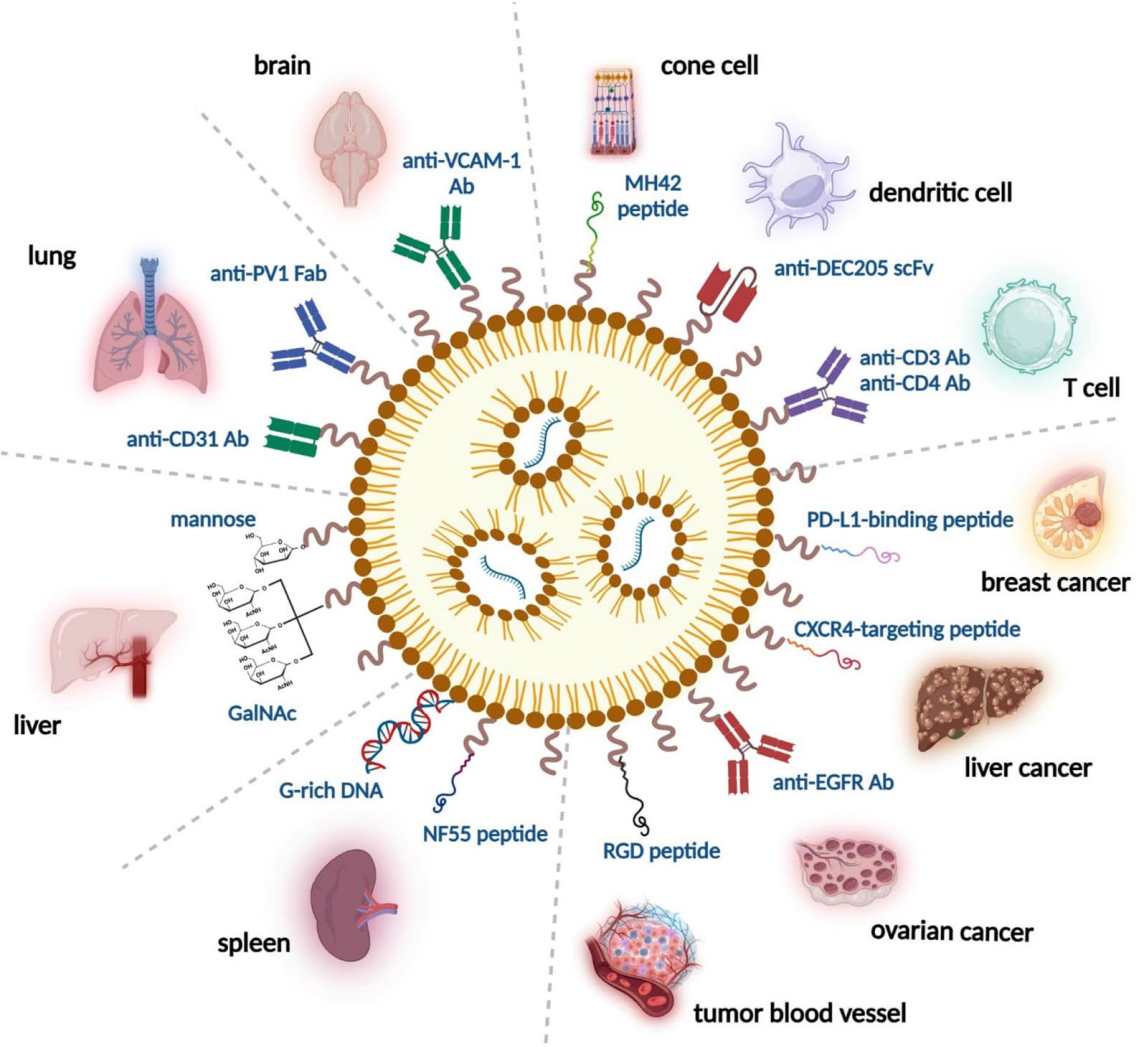
Strategies for ligand-mediated surface modification to achieve mRNA-LNP targeting. The surface of an mRNA-LNP can be modified with various ligands to enhance targeting specificity. Addition of sugars, nucleotides, peptides, antibody fragments or full-length antibodies can facilitate the targeted delivery of mRNA-LNPs to specific cells, tissues, organs or tumors. mRNA-LNPs with enhanced targeting specificity hold promise for improving the efficacy of mRNA-based drugs and vaccines. Created with BioRender.com

In recent decades, the targeted delivery of ligands that bind to TAAs (i.e., antibodies, peptides, polysaccharides and aptamers) has grown into a mature technology for cancer therapy. Novel specific ligand may be discovered by several different methodologies for identifying molecular recognition elements, such as phage display and systematic evolution of ligands by exponential enrichment (SELEX) [191, 239–241]. Such ligands have been reported to bind a variety of targets, including HA, folate receptor, transferrin receptor, CD44, EpCAM, c-Met, EGFR, HER2 and PD-L1, among others [242–248]. When considering targeting moieties, peptides and small molecules are typically simpler to manufacture than antibodies. However, antibody-based targeting provides the highest level of cell specificity and has been extensively explored in different therapeutic settings. Moreover, the use of antibody derivatives (e.g., scFvs or Fabs) may be appealing, as it can reduce the potential for host-versus-drug responses stimulated by Fc domains. The application of these targeting ligands for directing mRNA-LNPs to tumor cells is still a relatively new pursuit, and few studies on the topic have been completed. Nevertheless, some progress has been made in evaluating polysaccharide- or antibody-mediated delivery of RNAs to tumors. Although some off-target transfection in organs like the spleen and the liver may occur, ligand-based targeting of mRNA-LNPs appears to be an overall promising approach (Fig. 5).

Recent publications provide several successful examples of this approach. For instance, nanoparticles with CXCR4 chemokine receptor-targeting peptide, CTCE, and loaded with p53 mRNAs (CTCE-p53 NPs) showed active targeting of C-X-C Motif Chemokine Receptor 4 (CXCR4)-expressing RIL-175 murine hepatocellular carcinoma cells. When administered by intravenous injection, the combination of CTCE-p53 NPs with anti-PD1 monoclonal antibody induced marked regression of established RIL-175 tumors. This effect was achieved through the restoration of P53 protein levels and reversal of the immunosuppressive tumor microenvironment [249]. In addition, LNPs conjugated with PD-L1-binding peptides selectively delivered mRNA encoding phosphatase and tensin homolog (PTEN) to triple-negative breast cancer (TNBC) cells, which effectively induced anticancer immune response and reduced tumor growth in orthotopic and metastatic models [22]. In another study, LNPs decorated with anti-EGFR antibodies and loaded with Cas9 mRNA and PLK1 sgRNA were injected intraperitoneally into mice bearing disseminated ovarian tumors. The LNPs were efficiently taken up by the ovarian tumors, and gene editing of the PLK1 locus occurred within the tumor cells. Consequently, tumor growth was suppressed, leading to an extension in survival of the mice [196].

RGD is the fibronectin tripeptide binding domain that recognizes α_v_β_3_ and α_5_β_1_ integrins, which are respectively overexpressed in tumor-associated endothelium and tumor cells [250]. As such, RGD tripeptides have become a well-established targeting moiety for delivery of nanoparticles and biomaterials to tumor tissues. The use of RGD peptides for directing mRNA-LNPs toward different organs in vivo has also been documented [132]. In one study, Qin et al. developed 20 RGD-modified ionizable lipids and formulated 20 corresponding unique LNPs. Notably, the 1A RGD-based LNP could be used to effectively deliver Cas9 mRNA and sgRNA into HepG2 cells, leading to knockout of GFP expression. In vivo*,* these RGD-based hybrid LNPs achieved comparable mRNA delivery to both the liver and spleen.

### Organ targeting

Although LNPs can be used to efficiently deliver nucleic acids in vivo, their preferential accumulation in the liver tissue poses a significant limitation. Strikingly, as much as 30–90% of systemically administered LNPs accumulate in the liver, which would constitute a severe off-target effect if treating non-hepatic diseases [251, 252]. In order to transition mRNA-LNPs from prophylactic to therapeutic applications, researchers must overcome the crucial challenge of delivering mRNA payloads to non-hepatic tissues. Considerable efforts have been devoted to guiding mRNA-LNPs to specific tissues, as selective targeting remains a key obstacle to the advancement of precision medicine and mitigation of off-target and adverse effects associated with RNA-based therapeutics [185, 186].

While non-targeting mRNA-LNPs tend to primarily accumulate in the liver after administration via intravenous, intramuscular and subcutaneous routes, targeting ligands may be used to more precisely direct LNPs to hepatocytes or other specific liver cell types. For example, one study utilized LNPs decorated with mannose ligands to deliver mRNA encoding epitope peptides of Arachishypogaea protein 2 (Ara h2), a primary allergen in peanuts, to LSECs [253]. In C3H/HeJ mice sensitized to and subsequently challenged with crude peanut allergen extract, the prophylactic administration of the LSEC-targeted mRNA-LNP could inhibit anaphylaxis. This effect was mediated by suppression of Th2-mediated cytokine production, IgE synthesis and mast cell release of inflammatory factors. In other work, antibodies against the endothelial cell surface marker, PCAM-1 (platelet-endothelial cell adhesion molecule-1; CD31) were utilized for LNP targeting [195]. Intravenous administration of anti-PCAM-1-conjugated mRNA-LNPs led to low levels of hepatic uptake accompanied by an approximately 200-fold elevation of mRNA delivery and 25-fold increase of protein expression in the lungs compared to non-targeted counterparts. An alternative method for selective delivery of mRNA-LNP to hepatocytes involves the utilization of multivalent GalNAc targeting ligands, which enhance uptake through endogenous ApoE/LDLR binding [23]. In studies conducted on mice and non-human primates, GalNAc-containing LNPs effectively delivered CRISPR mRNA and gRNA targeting *ANGPTL3* in a gene editing therapy.

Targeted mRNA-LNP delivery is also being developed for treatment of inherited retinal degeneration. A set of potential targeting peptides that bind to the neural retina was identified via in vivo biopanning of a heptameric peptide phage display library [214]. One of the identified peptides, MH42, exhibited exceptional binding and internalization activity in the 661w mouse cone photoreceptor cell line and in vivo. LNPs with surface decoration of MH42 were then used to effectively deliver Cre and GFP mRNAs to photoreceptors within the neural retina of mice and rhesus macaques.

Another type of targeting agent is cell-penetrating peptides (CPPs), which have been widely employed to decorate nanoparticles and enhance of cell entry. One interesting study revealed that certain CPPs (i.e., NF424, NF436, and NF55) can be used to promote delivery of luciferase mRNA cargo to extrahepatic tissues, such as spleen and lung [254]. In particular, NF55 facilitated mRNA expression in spleen dendritic cells. However, the mechanism of CCP-mediated spleen targeting was not revealed. Another spleen-targeting molecule utilized for surface modification of LNPs was developed by Sinegra et al. In their study, the authors showed that 3’-SH DNA with G-rich motifs could be used to target mRNA-LNPs to the spleen [255]. The proposed mechanism underlying enhanced uptake in spleen involves DNA sequences forming a G-quadruplex secondary structure, which is capable of binding to class A scavenger receptors and triggering endocytosis.

Several studies have evaluated strategies for targeting LNPs to lung tissue. Plasmalemma vesicle-associated protein (PV1) is a known caveolae-associated protein, and Li’s group found that the biodistribution of anti-PV1 antibody was mostly in lungs and kidney [256]. Therefore, the group utilized an anti-PV1 Fab (called C4) to help guide mRNA-LNPs to lung tissues [24]. Systemic administration of PV1-targeted mRNA-LNPs led to greatly enhanced delivery of mRNA to lung tissues. A 40-fold improvement in firefly luciferase protein expression was observed in the lungs, as compared with the non-targeted control LNP. During the COVID-19 pandemic, treatment of SARS-CoV-2-infected individuals with neutralizing mAbs exhibited remarkable effectiveness in lowering hospitalization rates [257]. Along these lines, researchers developed lung-selective mRNA-LNPs encoding a broadly neutralizing antibody (8-9D) that could be efficiently delivered and expressed in the lungs of mice [258]. This approach effectively suppressed viral invasion, providing prophylactic and therapeutic protection against authentic SARS-CoV-2 challenge in K18-hACE2 transgenic mice. Conventional SARS-CoV-2 antibody therapies do not have enhanced lung delivery. Therefore, the utilization of lung-targeting LNPs carrying mRNA for antibody expression significantly enhanced antibody titers within the lungs.

Drug delivery across the blood–brain barrier remains a formidable challenge. Marcos-Contreras et al. investigated the efficacy of anti-vascular cell adhesion molecule 1 (VCAM-1)-conjugated mRNA-LNPs in targeting the inflamed brain [21]. Compared to ICAM- and transferrin receptor 1-targeted LNPs, the VCAM-1-targeted LNPs demonstrated superior selective accumulation in brain tissues. Further, the VCAM-1-targeted LNPs were engineered to carry mRNA encoding a therapeutic thrombomodulin protein, and the induced expression of thrombomodulin alleviated TNF-α-mediated cerebrovascular edema. These studies on specific targeting of liver, spleen, lung and brain tissues demonstrate that targeted delivery of mRNA to different organs is likely to become a feasible approach for treatment of various diseases.

Overall, the development of targeting strategies for nucleic acid delivery holds great promise for improving the efficiency and specificity of drug delivery, opening up new possibilities for the treatment of various diseases.

## Conclusions and prospects of mRNA drugs

mRNA-based drugs hold great promise for treating many types of disease, including infectious diseases and different types of cancer. Interest in mRNA-based drugs drastically increased after 2019, as evidenced by substantial increases in the numbers of patents and publications on the topic. According to the Derwent Global Patent Data (Clarivate), a total of 1213 patents in the field of mRNA-based medicines (including therapy, vaccine and delivery systems) have been filed worldwide from 2020 to April 2024. This activity represents an almost 5.4-fold increase compared to the period spanning from 2011 to December 2019, prior to the outbreak of the COVID-19 pandemic. However, major challenges remain in ensuring the safe and efficient delivery of bioactive mRNA molecules. Efficient delivery is crucial to this modality, as mRNA is a large and fragile molecule that can be easily degraded in the body. Additionally, delivery systems should ensure that the mRNA efficiently reaches the target cells and is taken up to produce the desired therapeutic effects. Moreover, modifying the surface of LNP carriers may allow the drugs to evade the immune system and improve circulation time. For instance, PEGylation can be used to enhance the stability of nanoparticles. Limited immune system activation is also crucial for the success of mRNA therapies, as unwanted immune responses can lead to adverse effects and reduce treatment effectiveness. Current efforts are focused on the design of mRNA sequences that are less immunogenic, and development of optimized LNP delivery systems to minimize immune reactions and increase cargo expression. The successful development of safe and effective delivery methods for mRNA-based drugs will require multidisciplinary studies, combining expertise in molecular biology, chemistry, materials science and immunology. Ongoing research aims to address the remaining challenges related to mRNA-based drugs in order to unlock the full potential of this emerging class of therapeutics.

At the end of 2023, the US FDA approved the first gene therapy using CRISPR/Cas9 for the treatment of stickle cell disease [26]. Exagamglogene autotemcel (Casgevy™) consists of non-viral, ex vivo gene-edited autologous CD34 + hematopoietic stem cells with reduced *BCL11A* expression that enhances fetal hemoglobin expression levels [259]. CPISPR technology can target mutations to specific genomic sites, and can potentially be utilized to create new treatments for a wide range of conditions, such as high cholesterol and leukemia. There are currently several phase I clinical trials underway to assess feasibility and safety of CRISPR-based therapeutics [260, 261]. VERVE-101 is the first trail on a base-editing treatment to inactivate expression of the *PCSK9* gene for the purpose of reducing LDL levels [262]. In another application, base-editing is performed on CAR7 T cells for treatment of leukemia. The CAR7 T cells are first made from healthy donor T cells by lentiviral transduction of a CAR that specifically targets CD7 + leukemia cells. Base editing is then performed to inactivate gene expression of CD52, CD7 receptor and the chain of the αβ T-cell receptor via non-viral delivery of codon-optimized BE3 mRNA and three sgRNAs [261]. The use of non-viral vectors for delivery of CRISPR-Cas9 can reduce off-target effects and provide greater safety compared to viral vectors. In another application, Foss et al. utilized amphiphilic peptide-mediated ribonucleoprotein delivery of CRISPR for genome engineering with a homology-directed repair template for a CAR. The resultant CAR T cells exhibited antitumor activity in a xenograft mouse model [263]. In addition to delivery of nucleic acids to cells in culture, LNPs are also suitable to deliver different CRISPR components (e.g., Cas9 and sgRNAs in plasmid DNA, mRNA or ribonucleoproteins; RNPs) to target tissues [264]. In one study, LNP delivery of Cas9 mRNA and sgRNA was evaluated in terms of its safety profile and showed no off-target effects, liver toxicity or Cas9-mediated immune responses [265, 266]. The encapsulation of CRISPR-Cas9 RNPs in modified ionizable LNPs has recently been shown to be a feasible approach for in vivo targeted delivery. Using this strategy in the context of cancer therapy, DNA editing activity could be directed to target tissues by conjugated antibodies on the LNP surface [196, 267].

The first clinical trial using LNP-encapsulated CRISPR-Cas9 was initiated in 2020 and targeted the *TTR* gene in hepatocytes for the treatment of transthyretin amyloidosis with cardiomyopathy. At present, this therapy is under evaluation in a phase III clinical trial (NCT06128629). This type of in vivo genome editing is expected to be a major focus of future therapeutic development efforts, as it has benefits of low cost, minimized genotoxicity and widespread potential for application.

Novel mRNA-LNPs hold great potential for targeting immune cell populations. The targeted delivery of mRNA to immune cells can help to reduce side effects associated with off-target mRNA delivery and enhance mRNA uptake efficiency. While there are several potential targeting strategies under evaluation, each has limitations that may hinder its clinical translation. For example, generating selective targeting ligands to attach on the surface of specific cell types is a complicated and time-consuming endeavor. Perhaps phage display libraries could be used to assist in screening and identification of ligand-receptor combinations. It is also likely that in coming years, utilization of AI tools will streamline the prediction of targeting ligands and accelerate the pace of development to allow for more clinical studies on targeting mRNA-LNPs. Along these lines, ML (machine learning) was used by Daniels et al. to predict the optimal combination of CAR signaling motifs for treatment of an in vivo tumor model [268]. Furthermore, ML technology can already facilitate prediction of antibody binding sites and activities in the development of LNP-mRNA targeting antibodies [149].

Numerous studies have shown that mRNA can effectively reach various organs, including the liver, lungs, spleen, eye and brain. As research progresses, the potential for reaching even more organs is expected to grow. However, it cannot be overlooked that LNPs have common drawbacks of immunogenicity and toxicity. Strategies such as incorporation of biodegradable materials, optimization of dosages, and identification of novel lipid alternatives may offer solutions to these challenges.

In this work, we have extensively reviewed many of the most prominent mRNA-LNP technologies utilized for cancer therapy. These technologies include gene editing, restoration of tumor suppressor genes, and cancer immunotherapy. The potential for in vivo expression of antibody therapeutics, such as neutralizing antibodies, bispecific antibodies and immunotoxins, is bolstered by mRNA-LNP delivery. This core technology is bringing about a new era in cancer treatment. Traditional antibody production is complex and time-intensive, requiring the development of stable cell lines and large-scale production under Good Manufacturing Practice (GMP) conditions. Leveraging mRNA-LNPs for in vivo antibody production holds enormous potential to overcome these obstacles and offers a promising avenue for advanced cancer therapy.

## Data Availability

All the data and materials supporting the conclusions were included in the main paper.
